# Ion Pairs for Transdermal and Dermal Drug Delivery: A Review

**DOI:** 10.3390/pharmaceutics13060909

**Published:** 2021-06-20

**Authors:** Mignon Cristofoli, Chin-Ping Kung, Jonathan Hadgraft, Majella E. Lane, Bruno C. Sil

**Affiliations:** 1 School of Human Sciences, London Metropolitan University, 166-220 Holloway Road, London N7 8DB, UK; b.dasilvasildossantos@londonmet.ac.uk; 2 School of Pharmacy, University College London, 29-39 Brunswick Square, London WC1N 1AX, UK; c.kung@ucl.ac.uk (C.-P.K.); jonathan.hadgraft@btinternet.com (J.H.); m.lane@ucl.ac.uk (M.E.L.)

**Keywords:** ion pair, topical, counter ions, partition, permeation, porcine, human, skin

## Abstract

Ion pairing is a strategy used to increase the permeation of topically applied ionised drugs. Formation occurs when the electrostatic energy of attraction between oppositely charged ions exceeds their mean thermal energy, making it possible for them to draw together and attain a critical distance. These ions then behave as a neutral species, allowing them to partition more readily into a lipid environment. Partition coefficient studies may be used to determine the potential of ions to pair and partition into an organic phase but cannot be relied upon to predict flux. Early researchers indicated that temperature, size of ions and dielectric constant of the solvent system all contributed to the formation of ion pairs. While size is important, this may be outweighed by improved lipophilicity of the counter ion due to increased length of the carbon chain. Organic counter ions are more effective than inorganic moieties in forming ion pairs. In addition to being used to increase permeation, ion pairs have been used to control and even prevent permeation of the active ingredient. They have also been used to stabilise solid lipid nanoparticle formulations. Ion pairs have been used in conjunction with permeation enhancers, and permeation enhancers have been used as counter ions in ion pairing. This review attempts to show the various ways in which ion pairs have been used in drug delivery via the skin. It also endeavours to extract and consolidate common approaches in order to inform future formulations for topical and transdermal delivery.

## 1. Introduction

The application of drugs via the skin provides a number of benefits. These include the potential to provide a steady-state release [1], the avoidance of first-pass metabolism in the case of transdermal formulations and the localisation of application for topical formulations [2]. Furthermore, the capacity for rapid cessation in cases of adverse reactions, the avoidance of side effects related to oral administration [3] and, with particular reference to non-steroidal anti-inflammatory drugs, the avoidance of gastrointestinal and renal problems [4] all contribute to the advantages of this delivery route.

Notwithstanding the many benefits, this mechanism of drug delivery needs to ensure that appropriate quantities of the drug are transported to the target site. The skin, however, does not offer an easy passage for the delivery of drugs. The outermost layer, known as the stratum corneum (SC), provides such an effective barrier that in the case of many topical formulations, only approximately 1–2% of the applied drug or active ingredient permeates [5].

This is partly due to the majority of pharmaceutical compounds being either weak acids or bases, with concomitant low aqueous solubility. As the most prevalent drug delivery system is oral, drugs are frequently developed as salts that ionise at physiological pH in an effort to facilitate processing and increase bioavailability. This poses a problem for topical and transdermal drug delivery, because of the lipid-rich SC that prevents the passage of such ionised drugs at therapeutic rates. In striving to increase and also to regulate the permeation of drugs, a number of different methods have been explored. These can be divided into two groups, namely active and passive. Active methods are those that use physical means to overcome barriers, such as iontophoresis, phonophoresis and microneedles [6]. Conversely, passive methods rely on the optimisation of the formulation. Such methods include increasing the thermodynamic activity of drugs in the formulation [7], the use of skin penetration enhancers [8] and also the use of ion pairs [9].

This review considers the research pertaining to the use of ion pairs in topical and transdermal deliveries. As early permeation studies highlighted problems associated with the use of rat, mouse and snake skin, such studies have been omitted [10,11]. In order to obtain data as best aligned to human skin responses as possible, permeation data are drawn exclusively from studies utilising human or porcine membranes [12].

## 2. Ion Pairs

### 2.1. Background

Ion pairing results when individual ions behave as a neutral species or group via electrostatic interactions, without creating any ionic or covalent bonds [13]. In general, this occurs most favourably in solvents with low dielectric constants (ε), as the electrostatic interaction between ions has less competition from potential interactions with the solvent system [14]. Ions in an aqueous environment may potentially mask their charges via electrostatic interactions, allowing them to partition more readily into a lipid environment, such as the SC, than individual ions [15,16].

The earliest theories concerning interactions between ions in electrolyte solutions included Milner’s complex statistical calculation of the distribution of ions in solution, which considered both electrostatic interaction and thermal motion. Another attempt to calculate the energy of the interaction between ions was undertaken by Ghosh in 1918. The author, however, assumed that ions adopted a rigid crystal-like arrangement, ignoring the impact of thermal motion. A significant advance in the area was made by Debye and Hückel in 1923 which proposed the interaction of a central ion (m) and other ions in solution. The concept of an ionic atmosphere or cloud was proposed by the authors suggesting that the relationship between the central ion and its neighbouring ions of opposite charge was a continuum, rather than a separate interaction.

The role of electrostatic interaction between pairs of ions was first considered by Semenchenko in 1924, but it was Bjerrum who developed the concept of ion pairs. It was suggested that at short distances, rather than the random thermal motion of ions in the ionic atmosphere, electrostatic interactions may develop between the central ion, m, and an oppositely charged ion, j. These interactions are sufficiently strong to overcome the independence of the individual ions, resulting in single-unit behaviour. When the two ions have the same number of charges, they will be considered electrically neutral. The formula for the total charge of two ion pairs was then devised according to Equation (1):

(z_j_ − z_m_)Q^0^ (1)

where:

z_j_ = charge number of the ion,
z_m_ = charge number of the central ion, and
Q^0^ = elementary electric charge 1.602 × 10^−19^ C (C refers to the differential double layer capacity with the units µF·cm^−2^).

As theorised above, the electrostatic energy of attraction between ions needs to exceed their mean thermal energy, allowing them to draw together and attain a critical distance, resulting in ion pair formation. As the pair is linked by electrostatic forces, but not ionic or covalent bonds, the lifespan of the pairing, while greater than the contact of mere individual collisions, is susceptible to collapse during strong collisions with other particles [17].

In Untersuchungen über Ionenassoziation which can be translated as “Studies of Ion Association” [18], Bjerrum describes the activity of ions in solution using different solvents. The author showed how the association or disassociation of ions is dependent on three main factors: ε of the solvent, temperature and size of the ions [19]. However, the reliability of Bjerrum’s theory was limited, as it did not account for elements such as solvation, concentrated solutions, solvents of high εs and hydrogen bonding [17,19,20,21]. Whilst extensions to the theory have been discussed [22], many of its basic principles are still applied for the purposes of topical and transdermal drug applications.

### 2.2. Ion Pairs in Topical and Transdermal Drug Delivery

#### Partition Coefficient

**Figure 1:**
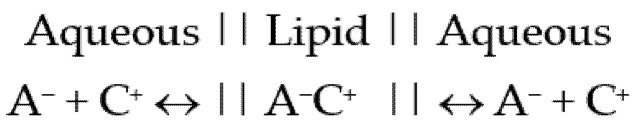
Ions in the aqueous phase moving into the lipid phase as an ion pair.

The appeal of ion pairs relates to the potential for compounds that are normally soluble in an aqueous environment, having their charges masked and enabling the partition of these systems into a subsequent lipid environment. A simple model of ion pairing would suggest that following passive diffusion into a lipid environment, the ion pairs would diffuse from areas of higher concentration to areas of lower concentration. When diffusing into an aqueous environment, the interactions with this more polar environment, comprising a higher ε, may reduce the degree of association between the ion pair, as suggested by Figure 1 below.

This simple overview, however, does not account for interactions with biological membranes, different aqueous solubilities of compounds, solvent system combinations, size differences in the ion pairs and other extrinsic factors. It does, however, suggest the use of simple partition experiments to determine the potential of ion pairs to partition from aqueous into lipid environments. Such experiments have long been considered representative of hydrophobicity, an essential component of quantitative structure—activity relationships (QSARs) used to predict skin permeability [23,24,25].

These tests and their results are sometimes referred to as partition coefficients, apparent partition coefficients or distribution coefficients. Additionally, the logarithm of these values may be provided and are then referred to as Log P, Log P apparent or Log D. For the purposes of simplicity, this review will make reference to a partition coefficient (PC) or PC studies and, where possible, will include the pH at which these experiments were conducted.

In general, PC measures the concentration of molecular species (S) in a non-miscible octanol (O) and aqueous (W) environment, as demonstrated in Equation (2), such that:

(2) $$\mathrm{PCs}\;=\frac{{\left[S\right]}_{O}}{{\left[S\right]}_{W}}$$

When incorporating factors such as pH and association or dissociation constants of compounds, a more representative formula for the PC of the chemical species is shown in Equation (3).

(3) $${\mathrm{PC}}_{S}=\frac{\left[U\right]+{\left[I^{-}]+[I^{-}I^{+}\right]}_{O}}{\left[U\right]+{\left[I^{-}]+[I^{-}I^{+}\right]}_{W}}$$

where:

U = unionised compound,
$I^{-}$= ionised version of the compound (anion),
$I^{+}$= counter ion (cation), and
$I^{-}I^{+}$= ion pair.

A typical example for the description of a weakly acidic compound states that there may be components of ionised, unionised and ion paired species in both the octanol and water phases. The same would hold for basic compounds that would use anionic counter ions.

PC experiments were used by Inagi et al. [26] to explain how indomethacin (IND), an acidic compound with an acid dissociation constant (pK_a_) of 4.5 [27], was able to permeate to a greater extent than the predicted pH partition theory value would suggest. The PC of IND was tested singularly, at different pH values (2.0–9.0), and in conjunction with various cations. It was found that the PC for IND decreased from ~ 1.6 × 10^3^ to 0.86 with the increase in pH (2.0–9.0). However, at a pH of 8.3, the PC of IND increased (~1.6 × 10^1^–~1.7 × 10^3^) with the increased concentration of sodium cations (0.02–0.10 M). Conversely, an increase in IND concentration, while maintaining a constant concentration of sodium cations, did not change the PC. The authors stated that this suggested the formation of ion pair complexes between IND and sodium cations, but no association between IND molecules. Using similar experiments, they concluded that IND was able to form ion pair complexes with ammonium cations, potassium cations and triethanolamine [26].

**Figure 2:**
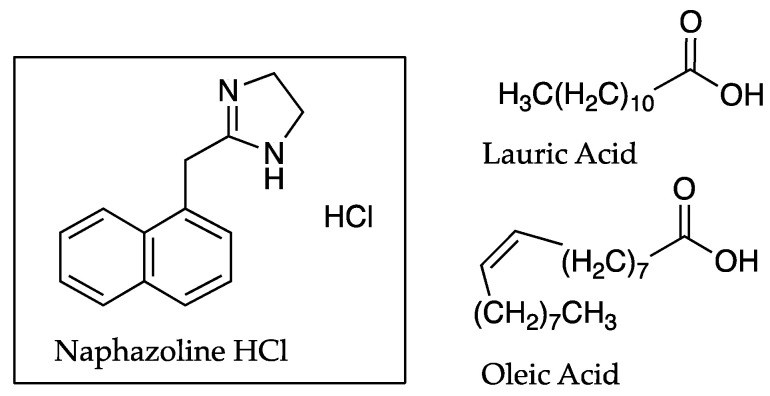
Naphazoline HCl and the counter ions LA and OA.

PC studies have been used by many authors in advance of, or in conjunction with, ion pair permeation studies. PC studies undertaken by Green et al. [28] indicated the ability of the strong base naphazoline hydrochloride (HCl) to form ion pairs with fatty acids, and these were reflected by an increase in permeation through human skin. As shown in Table 1, the PC value for naphazoline HCl at a pH of 7.4 was 0.02 ± 0.01, increasing to 0.36 ± 0.04 and 0.45 ± 0.06 with the addition of oleic acid (OA) and lauric acid (LA), respectively. A PC value was reported at pH 8.0 only for neat naphazoline HCl (0.03 ± 0.01), as the increased pH caused emulsification when fatty acids were present. The permeability coefficients determined from the skin permeation studies, where the donor solution was maintained at pH 8, were approximately 0.33, 2.17 and 2.58 cm h^−1^ × 10^−3^ for naphazoline HCl, and in conjunction with OA and LA [28]. The structures of naphazoline and the fatty acid counter ions are shown in Figure 2.

**Figure 3:**
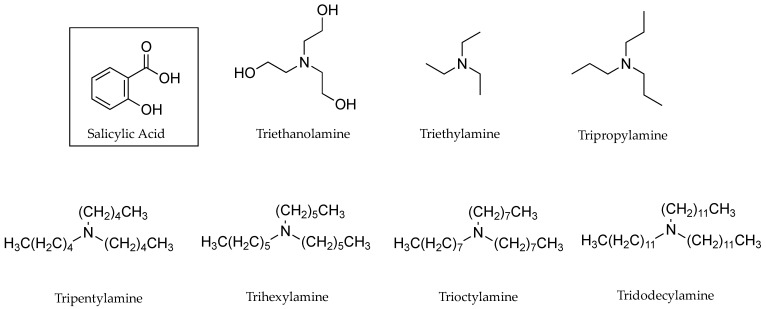
Salicylic acid the counter ions, triethanolamine, triethylamine, tripropylamine, tripentylamine, trihexylamine, trioctylamine and tridodecylamine.

Positive linear relationships were also found between experimental PC values of lignocaine HCl (0.19, 0.40 and 6.76) and corresponding flux values (1.2 ±1.2, 13.0 ± 2.0 and 118.0 ± 30.0 µg cm^−2^ h^−1^) through human skin, as seen in Table 2 [29]. Table 3 shows the results of PC experiments at pH values 5.0, 6.0 and 7.0 for benzydamine HCl. These indicate increasing PC (1.62, 5.75 and 28.18) and flux values (5.15 ± 2.42, 39.07 ± 10.50 and 269.09 ± 10.50 µg cm^−2^ h^−1^) in human skin permeation experiments as the fraction of unionised drug increased with increasing pH (6.3 × 10^−3^, 0.00631 and 0.627) [30]. Megwa [31] also reported that increases in PC results for the tertiary amine counter ions used in conjunction with salicylic acid (SA), shown in Figure 3, lead to increased permeation in human epidermis studies as depicted in Table 4.

**Figure 4:**
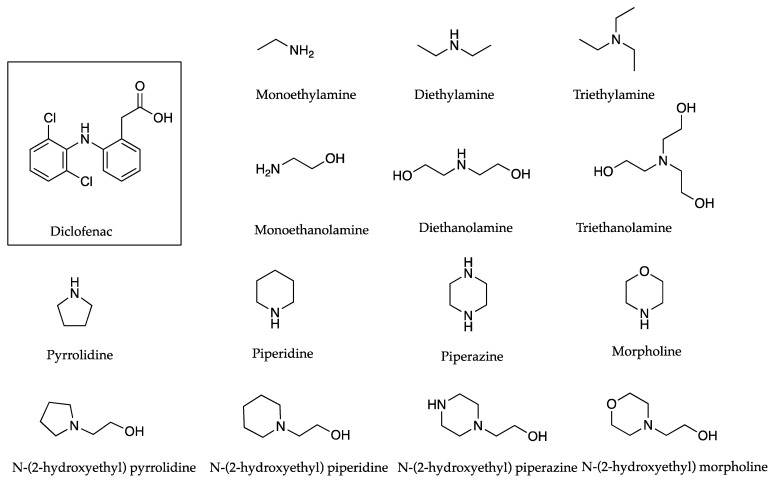
DIC with structurally related counter ions monoethylamine and monoethanolamine; diethylamine and diethanolamine; triethylamine and triethanolamine; pyrrolidine and N-(2-hydroxyethyl) pyrrolidine; piperidine and N-(2-hydroxyethyl) piperidine, piperazine and N-(2-hydroxyethyl) piperazine; and morpholine and N-(2-hydroxyethyl) morpholine.

In investigating the effects of counter ions on the permeation of diclofenac (DIC) through porcine membranes, it was determined that when comparing structurally related counter ions, a higher PC often corresponded to a higher permeation coefficient, as indicated in Table 5 below [32]. The only exception related to monoethanolamine and monoethylamine, where the PC values of 1.2 and 1.02 resulted in permeation coefficients of 0.70 and 2.00 cm h^−1^ × 10^3^. Chemical structures of DIC and the related counter ions are depicted in Figure 4.

**Figure 5:**
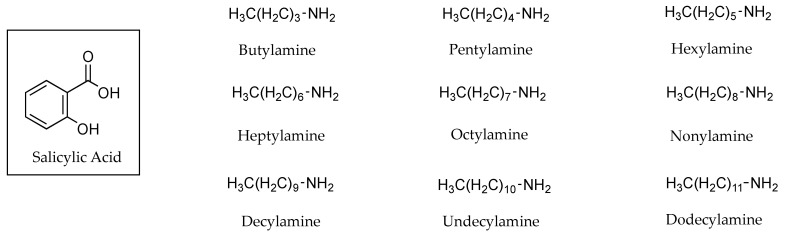
SA and primary amines with alkyl chains longer than four carbons, namely butylamine, pentylamine, hexylamine, heptylamine, octylamine, nonylamine, decylamine, undecylamine and dodecylamine.

PC has, however, not always correctly reflected the results of permeation studies [33]. No such correlation was found when counter ions were quaternary amines [31] or primary amines [34]. In the latter case, PC studies for SA using octanol and water, as opposed to isopropyl myristate (IPM) and water, toluene and propylene glycol (PG) or IPM and PG, indicated that PC values increased when SA was combined with primary amines that included alkyl chains longer than four carbons (as shown in Figure 5). PC values were reported for neat solutions of SA (0.550 ± 0.104) and SA in conjunction with butylamine (1.710 ± 0.108), pentylamine (5.170 ± 1.350), hexylamine (11.220 ± 1.014), heptylamine (17.950 ± 0.138), octylamine (24.340 ± 0.658), nonylamine (17.160 ± 0.416), decylamine (29.740 ± 4.330), undecylamine (22.010 ± 2.344) and dodecylamine (22.680 ± 3.452). These amounted to increases in PC values of SA from a minimum of 3 to a maximum of 54 fold. These increases in PC did not correlate with the results of human skin permeation data in which both flux values and permeability coefficients were lower than those determined for SA (0.89 ± 1.20 mg cm^−1^ h^−1^ × 10^−1^ and 8.9 cm h^−1^ × 10^−4^). One potential reason suggested by the authors was that SA was retained in the biological membrane used in the experiments.

**Figure 6:**
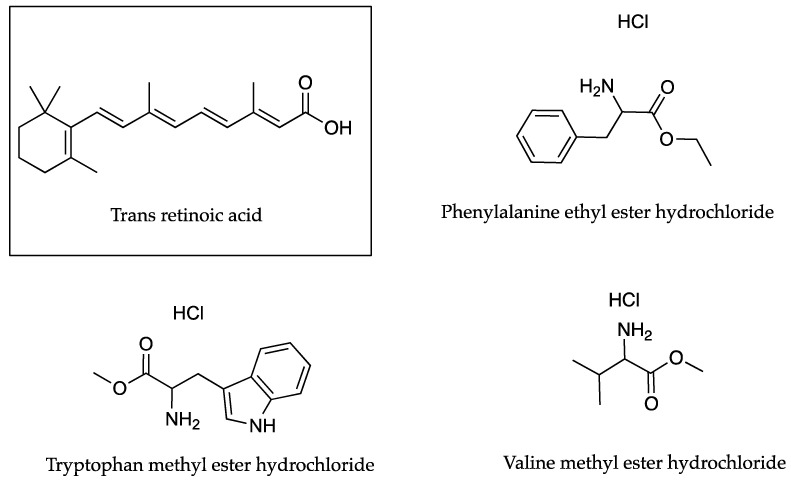
RA and phenylalanine ethyl ester hydrochloride, tryptophan methyl ester hydrochloride and valine methyl ester hydrochloride.

This was indeed the finding of Trotta et al. [35] who investigated the topical application of retinoic acid (RA) in conjunction with the methyl and ethyl esters of various amino acids as counter ions shown in Figure 6. These authors reported a linear relationship between PC results and skin accumulation in full-thickness pig ear skin. PC studies measured the partition of RA between IPM and an ethanol and pH 6.4 phosphate buffer (0.05 M) mixture, in the absence and presence of the abovementioned counter ions. A molar ratio of 1:50 (RA to potential counter ion) was used. The concentration of RA applied in the permeation experiments was 0.05% *w*/*w*. PC and skin accumulation (µg cm^−2)^ results were presented for neat RA (1.318 × 10^3^, 1.0 ± 0.2) and RA in conjunction with tryptophan methyl ester hydrochloride (1.259 × 10^4^, 2.3 ± 0.6) phenylalanine ethyl ester hydrochloride (3.090 × 10^4^, 3.4 ± 0.6) and valine methyl ester hydrochloride (4.467 × 10^4^, 3.7 ± 0.8). This relationship did not extend absolutely to skin flux values (µg cm^−2^ h^−1^) that were reported for RA (0.13 ± 0.02), and RA in conjunction with tryptophan methyl ester hydrochloride (0.19 ± 0.02), phenylalanine ethyl ester hydrochloride (0.23 ± 0.03) and valine methyl ester hydrochloride (0.21 ± 0.03), respectively. Here, the flux value for RA in conjunction with phenylalanine ethyl ester hydrochloride (0.23 ± 0.03 µg cm^−2^ h^−1^) exceeds that for the valine methyl ester hydrochloride (0.21 ± 0.03 µg cm^−2^ h^−1^), despite the PC results.

**Figure 7:**
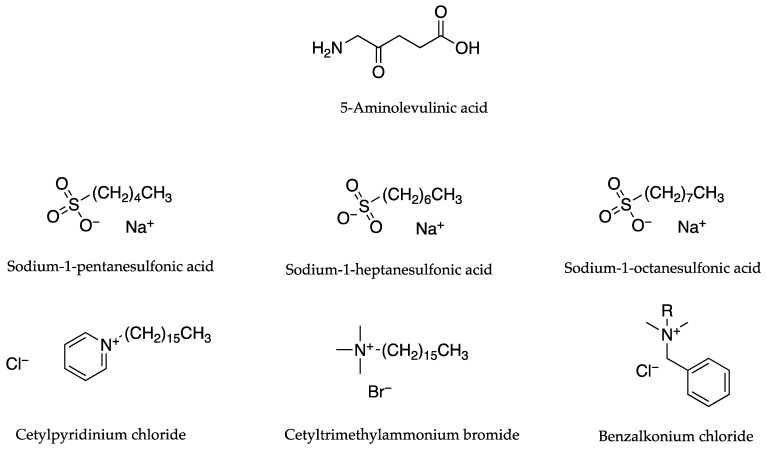
ALA and counter ions: sodium-1-pentanesulfonic acid, sodium-1-heptanesulfonic acid, sodium-1-octanesulfonic acid, cetylpyridinium chloride, cetyltrimethylammonium bromide and benzalkonium chloride.

Auner et al. [36] used PC studies to determine whether the lipophilicity of 5-aminolevulinic acid (ALA) could be increased by the addition of the various counter ions depicted in Figure 7. As ALA has two pK_a_ values, 4.0 and 7.9, pH values of 4.0 and pH 7.0 were employed. These resulted in an anionic charge dominating at pH 7.0 and a cationic charge dominating at pH 4.0. Results for PC studies, as seen in Table 6, showed that the lipophilic shift was higher in the case of pH 7.0 where ALA was tested alone (1.51), or in conjunction with cetylpyridinium chloride (CP) (9.12), cetyltrimethyl-ammonium bromide (8.13) and benzalkonium chloride (6.03). In the case of pH 4.0 where ALA was tested alone (1.20), or in conjunction with sodium-1-octanesulfonic acid (2.14), sodium-1-heptanesulfonic acid (4.68) and sodium-1-pentanesulfonic acid monohydrate (2.69), the lipophilic shift was lower. Cumulative permeation amounts of ALA through porcine skin (µg cm^−2^) after 4 h showed that, with the exception of cetyltrimethylammonium bromide, all anionic and cationic counter ions increased the cumulative permeation of ALA through porcine skin. ALA at pH 4.0, without a counter ion, resulted in a cumulative amount of 5.11 µg cm^−2^. This value increased with the addition of sodium-1-octanesulfonic acid (10.7 µg cm^−2^), sodium-1-heptanesulfonic acid (10.0 µg cm^−2^) and sodium-1-pentanesulfonic acid monohydrate (10.0 µg cm^−2^,) as counter ions. At pH 7.0 the cumulative amount of ALA alone was 6.5 µg cm^−2^. This increased to 11.0 µg cm^−2^ with CP as a counter ion and 7.0 µg cm^−2^ when benzalkonium chloride was the counter ion. As mentioned previously, the cumulative amount decreased with the addition of cetyltrimethylammonium bromide to 5.0 µg cm^−2^. There appeared to be no correlation between cumulative permeation and PC values.

In addition to the use of PC studies to test the partitioning abilities of counter ions, the factors described by Bjerrum as impacting their formation should not be disregarded. These included the size or radius of the counter ions as well as the ε of the solvent and temperature. A number of other areas have also been investigated in the literature such as the impact of the type of counter ions used, the influence of pH, the quantity of counter ions, the addition of permeation enhancers and the use of the latter as ion pairs.

### 2.3. Factors Influencing the Formation and Partition of Counter Ions

#### 2.3.1. Size and Type of Counter Ion

Bjerrum indicated that the distance between centres of charge when ions are paired is greater for large ions and smaller for smaller ions, thus potentially affecting the “cohesion” of the ion pairs. Fini et al. [15] tested the ability of a number of inorganic ions ranging from lithium to caesium cations to form ion pairs with the DIC anion, using PC as the main analytical tool. With the exception of lithium, the inorganic ions sodium, potassium, rubidium, and caesium were not as successful in forming ion pairs with the DIC anion. An inverse correlation was indeed identified between the size of the ionic radius and the PC. Lithium, with the smallest ionic radius, exhibited the highest PC value which reduced with increasing ionic radius of the inorganic ions: PC of lithium > sodium > potassium > rubidium > caesium, while the ionic radius of lithium < sodium < potassium < rubidium < caesium [15].

In addition to the influence on potential cohesion, smaller molecular size (as determined by molecular volume or molecular weight) is also a factor recognised by QSARs as a contributor to increased permeability coefficients [23,24,25]. In spite of this, Megwa et al. [34] found that a longer chain length of tertiary alkylamines used as counter ions in conjunction with salicylic acid (SA) resulted in improved permeation results through human skin, as shown in Table 7. Structures for these counter ions are shown in Figure 3.

The authors attributed this to the increase in lipophilicity as the alkyl chain length increased. It should also be noted that the highest molecular mass of any of the counter ions was 522 Da. While this did not appear to conform with the aforementioned rules relating to molecular size, it did result in the highest flux value. The same study also examined the effect of quaternary ammonium compounds on the permeation of SA and found that these did not impact the overall permeation of the active ingredient. It was determined that amine counter ions affect permeation through the human epidermis in the following sequence: quaternary < primary < secondary < tertiary.

The previously discussed Auner study [36] also examined the efficacy of various quaternary ammonium compounds as counter ions for ALA. As was noted, and can be seen in Table 6, these yielded diverse flux results in permeation tests using porcine skin. When paired with cetyltrimethylammonium bromide, despite having higher PC values than neat ALA (~8.13 × 10^−1^ in contrast to ~1.51 × 10^−1^), it was reported to have a cumulative permeation of approximately 5 µg cm^−2^ after 4 h, while the flux of ALA without the counter ion was approximately ~6.5 µg cm^−2^ for the same period. Benzalkonium chloride (PC value of ~6.03 × 10^−1^) and CP (PC value of ~9.12 × 10^−1^) did improve the cumulative flux values with results of approximately 7 and 11 µg cm^−2^, respectively, after 4 h [36]. A number of reasons for the permeation results were suggested by the authors, in accordance with studies by Takacs-Novak and Szasz [37] that examined the partition of quaternary ammonium substances. These included the influence of lipophilicity, size and flexibility, expressed as the number of rotating carbon-carbon sigma bonds, of the counter ions. Whilst it was mentioned that no significant effect had been attributed to the type (such as aliphatic or aromatic) of quaternary nitrogen compound, much importance was attributed to the lipophilicity and association constants of ion pairs, emphasising the necessity of attaining a critical separation distance, without which ion pairs cannot be formed.

The same study also tested the use of sulfonic acid sodium salts as counter ions in conjunction with ALA (Table 6). While there was an increase in the cumulative permeation of ALA at pH 4.0 from ~5.11 µg cm^−2^ h^−1^ when counter ions were added, there was no significant difference in the cumulative permeation amounts of the different counter ions. The use of sodium-1-octanesulfonic acid resulted in the highest cumulative permeation at ~10.7 µg cm^−2^ h^−1,^ while sodium-1-heptanesulfonic acid and sodium-1-pentanesulfonic acid monohydrate both had cumulative permeation results of ~10.0 µg cm^−2^ h^−1^.

**Figure 8:**
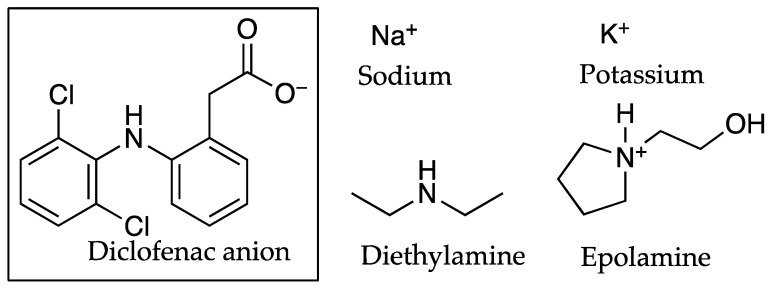
DIC anion with inorganic counter cations sodium and potassium and organic counter cations diethylamine and epolamine.

PC investigations indicated that organic cations were more successful than inorganic cations when forming ion pairs with the DIC anion [15]. It was suggested that when a salt contains both an organic anion and cation, some of the hydrophobic character remains. This was described by Minghetti and co-workers who considered the impact of four salts used in pharmaceutical applications in conjunction with DIC on the permeation of the latter through human skin. The salts were described as sodium, potassium, diethylamine and epolamine (N-(2-hydroxyethyl)pyrrolidine), the structures of which can be see in Figure 8. The findings suggested that associating an organic cation, such as the diethylamine and epolamine, and an organic anion in pharmaceutical salts guarantees improved permeation results. These results are contained in Table 10, showing the flux values for DIC (µg cm^−2^ h^−1^) in conjunction with the inorganic counter ions sodium (2.29 ± 0.37) and potassium 1.35 ± 0.72 increase with the organic counter ions diethylamine (5.60 ± 2.14) and epolamine (2.90 ± 0.91), when using water as a solvent [38].

As discussed previously, Trotta used a variety of amino acid esters to increase the delivery of RA, a hydrophobic molecule that is practically insoluble in water, to the skin. Skin accumulation results (µg cm^−2^) showed that valine methyl esters (3.7 ± 0.8) and phenylalanine ethyl esters (3.4 ± 0.6) were more successful than the tryptophan methyl esters (2.3 ± 0.6) in increasing the accumulation of RA in pig skin [35].

**Figure 9:**
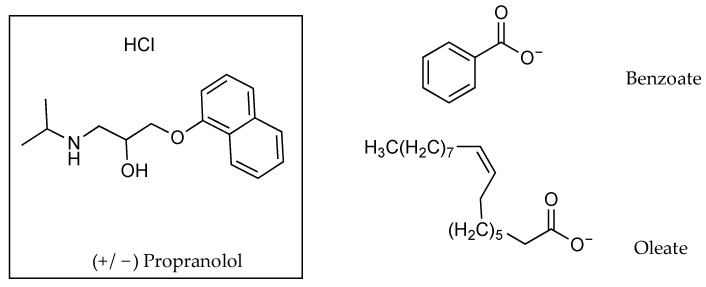
Propranolol plus counter ions benzoate and oleate.

Two counter ions, benzoate (Bz) and oleate (Ol), were also considered by Cilurzo and co-workers [33] for the pairing of a chiral compound, propranolol (PR), as a racemate, RS-PR, and as a single enantiomer, S-PR (Figure 9 and Table 8). Despite partition studies reflecting a 78% increase in the PC value for RS-PR as well as an 104% increase for S-PR, when tested with the Ol counter ion, permeation data showed drug fluxes reducing in conjunction with the counter ion. The flux of the racemate in saline reduced from 18.0 ± 5.1 µg cm^−2^ h^−1^ to 7.0 ± 1.4 µg cm^−2^ h^−1^ when combined with Ol. The flux of the racemate in mineral oil (MO) decreased from 24.2 ± 4.7 µg cm^−2^ h^−1^ to 7.3 ± 1.4 µg cm^−2^ h^−1^ when combined with Ol. The S-enantiomeric form of PR showed higher flux results than the racemate when applied neat, whether in saline (44.7 ± 5.1 µg cm^−2^ h^−1^) or MO (41.9 ± 1.5 µg cm^−2^h^−1^). When Ol was used as a counter ion, the flux of the S-enantiomer plummeted to 6.2 ± 0.9 µg cm^−2^ h^−1^ when in saline and to 11.1 ± 1.7 µg cm^−2^ h^−1^ when in MO. When Bz was used as the counter ion, it increased the PCs of the racemate and the S-enantiomer marginally, from 10.00 and 9.33 to 11.74 and 11.48, respectively. This again failed to translate into an increase in the flux from saline for both the racemate (18.0 ± 5.1 to 1.7 ± 0.2 µg cm^−2^ h^−1^) and the S-enantiomer (44.7 ± 5.1 to 3.0 ± 0.4 µg cm^−2^ h^−1^). The situation was repeated when Bz was used as the counter ion with MO was the solvent. The flux for RS-PR reduced from 24.2 ± 4.7 to 1.6 ± 0.3 µg cm^−2^h^−1^ and for S-PR from 41.9 ± 1.5 to 2.9 ± 0.7 µg cm^−2^ h^−1^ [33].

In 2012, Fini et al. [32] considered the ability of DIC salts in aqueous solutions to permeate through porcine membranes. The choice of counter ions (Figure 4) included aliphatic alkyl amines: mono, di and tri ethyl amines, which were contrasted with mono, di and tri ethanol amines. Examples also included a range of cyclic substituents: pyrrolidine, piperidine, piperazine and morpholine. These were contrasted with structurally similar molecules that included hydroxy ethyl side chains, namely N-(2-hydroxyethyl) pyrrolidine, N-(2-hydroxyethyl) piperidine, N-(2-hydroxyethyl) piperazine and N-(2-hydroxyethyl) morpholine. The solutions used in these studies were saturated, which made the comparison of flux values challenging. Instead, flux was divided by solubility (in µg/cm^3^) in order to obtain a permeability coefficient. When comparing structurally related pairs of salts (Table 5), the authors ascertained that the results suggested “that a common mechanism associated with permeation is the hydrophobicity of the permeant species”. In most cases, the salt which contained the hydroxy group showed a higher aqueous solubility (when compared to the paired ion that did not contain the same hydroxy substituent), but this generally did not translate into a higher permeation coefficient. Counter ions pyrrolidine, piperidine and piperazine resulted in increased permeation coefficients when compared to their related counter ions containing hydroxy groups. Morpholine counter ions showed an affinity for the aqueous phase which reduced permeation when compared to other cyclic related salts.

**Figure 10:**
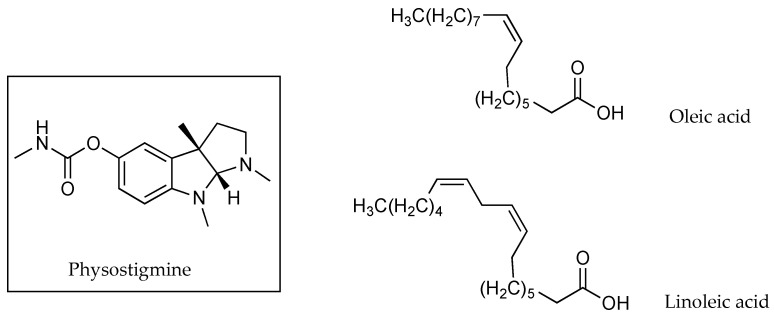
Physostigmine and counter ions OA and LA.

The impact of one and two double bonds on an 18-carbon fatty acid counter ion on the flux of physostigmine (PHY) through porcine skin, was described in 2005 by Wang [39]. OA (C18:1) and linoleic acid (C18:2), whose structures can be seen in Figure 10, resulted in PC values for PHY, between IPM and PG, of 0.225 ± 0.035 and 0.219 ± 0.01, respectively. Flux values were 19.7 ± 8.2 µg cm^−2^ h^−1^ for the counter ion C18:1 and only 2.4 ± 0.9 µg cm^−2^ h^−1^ when the counter ion was C18:2, despite solubility for the two being very similar (83.9 mg mL^−1^ for C18:1 and 85.3 mg mL^−1^ for C18:2). The authors suggested that the presence of the additional unsaturation may have caused the retention of the complexes in the SC lipids [39]. Conversely, the effect of a single unsaturation on the OA structure was also mentioned by Green et al. [28] as a potential reason for making this a better permeation enhancer than LA, a 12-carbon saturated fatty acid.

**Figure 11:**
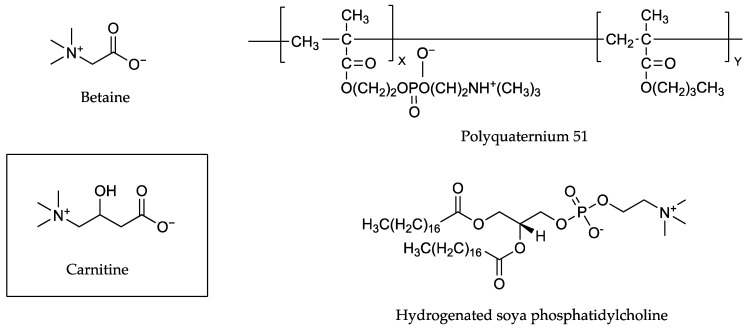
Carnitine with 3 different zwitterion counter ions: betaine, polyquaternium-51 and HSC.

More recently in 2019, In and co-workers [40] tested three different zwitterions in conjunction with carnitine Figure 11, which prevails as a zwitterion in weakly acidic or neutral pH solutions. Solutions comprising a 4% weight of carnitine, alone and in equimolar ratios with betaine, polyquaterium-51 or hydrogenated soya phosphatidylcholine (HSC), were used for permeation experiments. When combined with the active in solution, betaine and polyquaterium-51 reduced carnitine’s percutaneous penetration, while the combination with hydrogenated soya phosphatidylcholine (HSC), a higher-molecular-mass (762.1 Da) long-chain-carbon structure, nearly tripled it. The percentage of applied carnitine found in epidermal layers of porcine skin after 24 h was approximately 9.13% when carnitine was applied without any counter ion, 4.25% when applied with betaine, 6.55% when applied with polyquaterium-51 and 23.71% when applied with HSC [40].

#### 2.3.2. Dielectric Constant (ε)

In 1956, Kraus [19] tested components of Bjerrum’s theory, including the relationship between the dissociation of ion pairs to the ε of the solvent. Using water–dioxane mixtures as the solvent system and the salt tetraisoamylammonium nitrate, it was found that for values of ε greater than 44, ions were dissociated. Tetrabutylammonium bromide and sodium bromate were found to be dissociated for values of ε above 50. Irrespective of whether aqueous or organic solvents were used, there was a clear relationship between a higher ε reducing the association between ion pairs, and a lower ε increasing the association between ion pairs.

The impact of solvents with different ε values may be tested by determining the conductivity of the ions in solution. The higher the quantity of ions in solution, the higher the conductivity. As ion pairing increases, charges become masked or neutralised, resulting in lower conductivity results [14]. The use of conductivity measurements can therefore be an important tool to test the impact of solvent adjustments on the formation of ion pairs. By increasing the proportion of a particular solvent with a lower ε, columbic interactions between the oppositely charged ions of the potential ion pairs may become more stable, resulting in the formation or stabilisation of ion pairs, and thus potentially facilitating partition.

**Figure 12:**
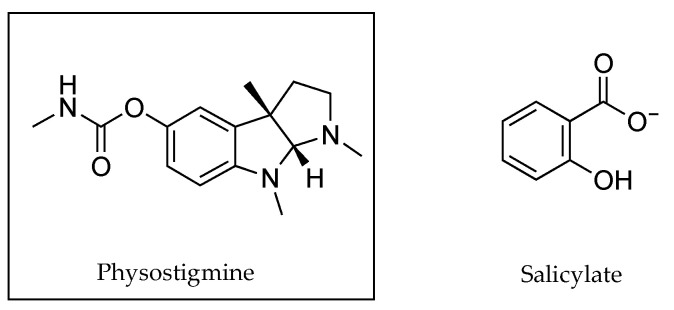
Counter ions PHY and Salicylate.

In the early 1990s, Pardo et al. [41] tested IPM, isopropyl alcohol (IPA) and various mixtures of both solvents to determine the impact of solvent adjustment on the permeation of counter ions PHY and salicylate (Figure 12) without the use of conductivity measurements. IPA’s ε, 18.62 at 20 °C [42,43], was far higher than the value determined for IPM, 3.31 at 25 °C [44]. Nonetheless, it was still only approximately one-quarter of the value of the ε of water, determined to be 80.37 at 20 °C [43]. As seen in Table 9, it was found that a solvent mixture comprising a 70:30 ratio of IPA to IPM had the best impact on permeation. Flux of PHY increased from 0.56 ± 0.08 × 10^4^ µmol cm^−2^ m^−1^ at its lowest rate determined when the solvent comprised 100% of IPA, to 44.27 ± 9.16 × 10^4^ µmol cm^−2^ m^−1^ when the solvent mixture contained 70% IPA. Flux of salicylate increased from 1.47 ± 0.11 × 10^4^ µmol cm^−2^ m^−1^ at its lowest rate, which occurred when the solvent comprised 100% IPM to 61.66 ± 2.54 µmol cm^−2^ m^−1^ when the proportion of IPM was reduced to 30%.

In early 2000, Megwa et al. [31] took a different approach. Instead of measuring the impact of different solvents on the conductivity of ions, the authors tested the conductivity of SA alone and in conjunction with a variety of potential counter ions in a fixed ethanol-propylene glycol (2:1 *v*/*v*) solvent combination in equimolar concentrations. As shown in Table 7, when the counter ions were tertiary amines, the conductivity of SA diminished with each consecutive increase in the length of the carbon chain of the counter ion. A relationship could be seen between the length of the carbon chain of the tertiary amine, conductivity, PC and flux: longer carbon chains resulted in lower conductivity, which generally represented higher PC values and higher flux, as seen in Table 4. However, this was limited to tertiary amines. The relationship did not extend to quaternary or primary amines [34],where, in the latter, there was very little difference in conductivity between neat SA or in conjunction with counter ions.

Four individual single solvents were tested with the inclusion of four different counter ions in conjunction with DIC by Minghetti et al. [38]. The solvents included water, PG, Transcutol^®^ (TC) and OA. The solubility of the drug which included its associated counter ion was generally much higher with TC and OA, yet flux values were greater when water and OA were used (Table 10). This research highlighted a key factor in ion pairing formulation, namely that high concentration of the drug does not guarantee an increase in flux values. The authors determined that the solubility parameters of the salts, as well as the solvents, TC and PG, were all comparable. They stated that “small differences in solubility parameters of a drug and its vehicle do not cause extensive partitioning of the drug out of its vehicle”. Unsurprisingly the flux values measured in TC and PG were the lowest among the four vehicles for all the four salts, as “the lower the tendency of the solute to leave the donor phase, the lower the flux.” These similar solubility parameters indicated that the solute might be more likely to be soluble in these solvents, but less likely to partition out of the vehicle. The authors concluded that permeability in permeation studies comprised two distinct features: firstly, the solubility of the solute in the donor phase and, secondly, the ability to partition out of the formulation and into the membrane.

In 2005, Wang and co-workers [39] investigated the impact of two single solvents, PG and mineral oil (MO), on the permeation of PHY in conjunction with fatty acid counter ions in porcine skin (Table 11). Conductivity measurements were undertaken in a manner similar to those employed by the Megwa [31] study mentioned above. The conductivity of PHY was tested alone and then in combination with a series of fatty acids of increasing carbon chain length in PG and then in MO. The main difference between the two studies was that the concentration of fatty acid: drug mixtures had a molar ratio of 50:1. Notwithstanding a low conductivity value for PHY in PG when tested in isolation, testing revealed a decrease in the conductivity of the PHY–counter ion solution, with an increase in the alkyl chain length of the fatty acid counter ion. These results for PHY unmixed and then combined with fatty acid counter ions of carbon chain lengths: 2, 3, 8, 10, 12, 18:1 and 18:2 were ~0.25, 16, 14, 10, 9.5, 8, 3 and 4 µS cm^−1^. Conductivity results were extremely low when conducted in MO. PHY alone in MO resulted in a conductivity value of ~9.25 × 10^−2^ µS cm^−1^ and never increased beyond ~9.4 × 10^−2^ µS cm^−1^ when combined with counter ions. The lack of any significant change in the conductivity of MO containing fatty acids when PHY was added, suggested to the authors that no ionisation of the PHY occurred, leading to an absence of ion pairing in this solvent system. Flux results were determined for saturated solutions of MO and PG. The counter ion with the longest carbon chain, OA (C18:1), consistently showed the best flux values in both solvents, 19.7 ± 8.2 µg cm^−2^ h^−1^ in PG and 13.9 ± 7.1 µg cm^−2^ h^−1^ in MO, as shown in Table 11. PHY together with the OA counter ion was greater than 5-fold more soluble in PG (83.9 mg mL^−1^) than in MO (15.9 mg mL^−1^). However, flux was only 1.4-fold greater. The lag time for the MO vehicle was ± 4.8 h whereas PG was shown to be ± 8.8 h.

Partitioning of PHY from PG was subsequently explained by the authors based on two factors. Firstly, the ion pairing that resulted in the reduced polar environment and secondly the impact of increasing lipophilicity due to the increased carbon chain length of the counter ion. Where ion pairs were formed with short chain fatty acids, these were described as strong ion pairs, due to a higher polarity of the counter ions. The longer the alkyl chain, the weaker the ion pairing, but the more lipophilic the nature of the ion pair. A more stable ion pair was noted to have lower lipophilicity than a more unstable one, making partitioning less likely. The authors also raised the importance of concentration as a driving force for drug diffusion as well as the impact of the solvent’s contribution to improving the partitioning of the drug into the skin.

As shown in Table 8, MO and a saline solution were used as potential solvents when using PR as a racemic mixture or in its S-enantiomeric form, in conjunction with counter ions. PC values increased due to ion pairing with Ol, from 10.00 and 9.33 for (RS)-PR and (S)-PR without Ol to 17.78 and 19.05 with Ol. Solubility also increased in MO from 1.321 and 3.111 mg mL^−1^ for (RS)-PR and (S)-PR without Ol to 3.930 and 5.874 mg mL^−1^ with Ol. Despite the increased PC and solubility values, when MO was used as the donor solvent in permeation experiments, flux values were comparatively low (7.3 ± 1.4 and 11.1 ± 1.7 µg cm^−2^ h^−1^ for (RS)-PR and (S)-PR in combination with Ol, versus 24.2 ± 4.7 and 41.9 ± 1.5 for (RS)-PR and (S)-PR without the counter ion). The authors concluded that this could be due to the stabilisation of the solute in the donor phase [33].

More recently, in 2019, conductivity was used in a novel fashion by In et al. [40], to determine the optimal molar ratio of zwitterionic ion pairs, with the lowest conductivity occurring at approximately a 1:1 ratio for the chosen counter ion. This resulted in an extension of previous approaches which considered the impact of the dielectric constants of solvents on the conductivity of ions in solution, or the impact on conductivity of using different counter ions.

#### 2.3.3. Temperature

Another factor introduced by Bjerrum in the late 1920s was the impact of temperature on ion pairing. In the paper “*Evolution of the Ion-Pair Concept*”, Kraus [19] explained that for solvents of a high ε value an increase in temperature caused an increase in ion pairing, whilst the converse is true for solvents with a low ε. Conductance measurements were used by Kraus as one possible method to demonstrate the increase in ion association in solvents of higher εs, with increasing temperature. The author showed that for solutions using solvents with a ε greater than ~10, and with a concentration range between 0.1 and 0.01 *N* the conductance values increase with increasing temperatures as a result of decreasing viscosity. As shown in Table 12, as temperature increases, the conductance increases at a decreasing rate. The conductance value reaches a maximum and then begins to decrease.

In addition, from Table 13, it is evident that the lower the ε, the lower the temperature of the conductance maximum, for a given concentration. Methylamine, ammonia and methanol have ε values of 9.4 at 25 °C, 22.4 at ~33.3 °C and 32.8 at 25 °C. Methylamine with the lowest ε has a conductance maximum at 15 °C. Ammonia, with a ε value approximately midway between methylamine and methanol, has its conductance maximum at 25 °C. Finally, methanol, with the highest ε of the three solvents, has a conductance maximum at 150 °C.

The author explained that as temperature rises, ion association increases until the decrease in conductivity, due to the ion association, just offsets the increase in conductivity, attributable to increasing fluidity, at the temperature of maximum conductance. From this temperature, ion association continues to increase and conductance to decrease. As the concentration of the solution is subject to very little variance due to changes in temperature under the specified conditions, the conductance provides a guide to the increasing association attributable to increasing temperature. It was further noted that the actual extent of ion association is greater than indicated by the conductivity measurements, as the fluidity of the solution increases with the temperature, causing the conductance values to be greater than expected.

#### 2.3.4. pH

As previously mentioned, pH modification in given solvent systems introduces changes to PC values. In this section, some of the concepts formerly mentioned will be revisited for the purpose of clarity and contextualisation of theory.

The pH partition theory suggests that only unionised drugs can permeate lipid membranes, whilst ion pairing attempts to achieve this via the masking of charges. In order to optimise partition and permeation of several molecular compounds, pH has been investigated in various respects. An example of this is the exploration of the slightly acidic pH values long attributed to the surface of the skin [45], and the physiological pH values associated with the lower layers of this biological membrane. Hadgraft et al. [46] tested whether such a pH gradient could be used to ionise the participating compounds, sodium salicylate and Ethomeen S12 (N,N-bis(2-hydroxyethyl)oleylamine), enabling the formation of ion pairs between the two. The purpose was to facilitate the transport of the active ingredient, salicylate, through the skin. Whilst this increased the transport of salicylate by approximately 3 fold when using a preparatory artificial lipid membrane and did not affect the control compound, caffeine (a weakly basic drug that was cationic at the pH values examined), the results could not be replicated when using human skin. Instead, the counter ion, Ethomeen S12, improved the transport of both salicylate and caffeine, suggesting an interference with the skin barrier.

The aforementioned studies by Green et al. [28] also involved the manipulation of pH. The purpose of such experiments was to facilitate the transfer of cations across human skin using ionised OA and LA as counter ions. In this study, however, the pH of the donor phase solution used in permeation experiments was higher than that of the receptor solution and also exceeded the pK_a_ of both LA and OA. The drug was basic in nature and ionised at the donor’s pH environment. Though the counter ions, LA and OA, increased the in vitro skin permeation of all the permeants, it was concluded by the authors that the increased flux of the cationic naphazoline could be partly attributed to ion pairing.

Valenta and co-workers’ research in the late 1990s [29] considered the permeation of lignocaine hydrochloride in human skin. These authors used a range of donor solutions with different pH values: 4.0, 5.5 and 7.0, intended to reflect various physiological conditions. As shown in Table 2, the flux was 1.2 ± 1.2 µg cm^−2^ h^−1^ at pH 4.0, 13.0 ± 2.0 µg cm^−2^ h^−1^ at pH 5.5 and 118.0 ± 30.0 µg cm^−2^ h^−1^ at pH 7.0. PC values of the lignocaine salt were 0.19 at pH 4.0, 0.40 at pH 5.5 and 6.76 at pH 7.0, both increasing with higher pH values. This was due to the increase in the amount of unionised base, consistent with the pH partition hypothesis.

Megwa and co-workers [34] demonstrated a procedure for determining the optimum pH conditions for ion pair formation between SA and various amines. The system employed was very similar to a typical PC study, with the use of toluene instead of octanol as the lipid phase, and a range of pH buffers (pH 2.5–7.5) as the aqueous phase. Following a comparable approach to that of Green [28], the drug was dissolved in the aqueous buffers and the counter ions added to the lipid phase, as opposed to dissolving the potential counter ions in the aqueous phase only. The optimal pH for the formation of ion pairs with the amines used (methylamine, ethylamine, propylamine, butylamine, diethylamine, triethylamine and triethanolamine) was ascertained to be pH 5.0.

Further studies involving SA were also undertaken by Smith and Irwin [47] in the same year. The authors tested both solutions and saturated suspensions of SA over a range of pH values. As hypothesised, experiments showed that the flux of SA across human skin from solutions was dependent on the pH of the vehicle (Table 14). The authors also determined that the flux from saturated suspensions across human skin remained relatively constant throughout the experiments (mean flux 1.09 ± 0.202 µmol cm^−2^ h^−1^) regardless of the different pH values (pH 1.84, 2.35, 2.80, 3.14, 3.45, 3.73, 4.17, 4.71). This confirmed that permeation is related to the concentration of unionised molecules, and is independent of pH or pH gradients (when a saturated suspension is used). Furthermore, this study demonstrated that the solubility of unionised SA in a suspension remained constant at different pH values, indicating a method of overcoming the impact of pH on solubility and maintaining maximum solubility [47].

A previously mentioned study by Auner et al. [36] involved devising optimal pH values for ion pairing which enabled the testing of both cationic (pH 7.0) and anionic (pH 4.0) counter ions in conjunction with ALA, a molecule that comprises two pK_a_ values (4.0 for the carbonyl group and 7.9 for the amino group). Analysis using PC studies indicated increased lipophilicity when ALA was combined with any of the counter ions. Higher lipophilicity resulted in increased permeation through porcine skin with all counter ions, with the exception of cetyltrimethylammonium bromide, as shown in Table 6.

A study by Sarveiya et al. [30] also tested the influence of donor compartment pH values in permeation experiments on the ionisation, partitioning and flux of benzydamine HCl, as seen in Table 3. In human skin studies, the authors found that as the PC increased with an increase in pH, so did the flux. At pH 5.0 PC value was 1.62 and flux 5.13 ± 2.42 µg cm^−2^ h^−1^; at pH 6.0 PC value was 5.75 and flux 39.07 ± 10.5 µg cm^−2^ h^−1^ and at pH 7.0 PC value was 28.18 and flux 269.09 ± 58.5 µg cm^−2^ h^−1^. The authors did suggest, however, that as the pH increased beyond these values, although the unionised fraction and the permeability coefficient would increase too, flux would be limited due to decreasing solubility.

**Figure 13:**
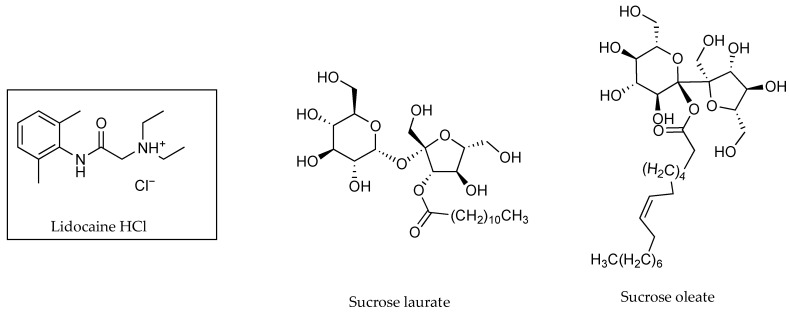
Lidocaine HCl with sucrose laurate and sucrose oleate.

In research by Cázares-Delgadillo [48] that investigated the impact of sucrose esters on the permeation of lidocaine HCl (see Figure 13) using porcine skin, both PC and permeation experiments were considered at pH values 5.0, 7.0 and 9.0. The fraction of unionised species was derived from the Henderson–Hasselbalch equation, and it was determined that at pH 5.0 lidocaine was completely ionised, at pH 7.0, 11% was unionised and at pH 9.0, 93% was unionised. The PC studies reflected this, with higher values attributed as pH increased (0.09 ± 0.002 at pH 5.0, 1.15 ± 0.007 at pH 7.0 and 1.17 ± 0.0043 for pH 9.0). Permeation of lidocaine alone also increased when the pH value of the buffered solution increased. As all solutions were saturated, permeability coefficients were obtained by dividing the flux by the concentration of the drug in the applied formulation. As shown in Table 15, permeability coefficients increased as pH increased. Apparent permeability coefficients for different pH values of lidocaine HCl and in the presence of sucrose laureate (L-TC) and sucrose oleate (O-TC) are also shown in Table 15. It was determined that pre-treatment of the porcine ear membranes with L-TC or O-TC in Transcutol^®^ had a surprising impact on flux, permeation coefficients and overall enhancement, as results deviated from those expected due to the abovementioned pH partition hypothesis. At pH 5.0 and pH 7.0, (where 100% and 89%, respectively, of lidocaine was ionised) L-TC enhanced the permeability coefficient of lidocaine alone by 11.95 and 10.84 fold. At pH 9.0, where lidocaine was 100% unionised, the presence of L-TC reduced the permeability coefficient to 59% of lidocaine HCl. Conversely, the application of O-TC (while increasing the permeability coefficient by 3.77 and 3.45 fold of that of lidocaine alone at pH values 5.0 and 7.0, respectively) led to an improvement in the permeability coefficient of the unionised lidocaine by 2.67 fold at pH 9.0. Investigations by the same group also indicated that L-TC causes lipid extraction and fluidisation of the skin barrier, creating structural disorder and an increase in micropores in the membrane which may facilitate greater penetration of the ions. The authors were, however, unable to rationalise the decreased movement of unionised lidocaine through the biological membrane. Whilst this may indeed suggest the formation of ion pairs at pH values at which lidocaine is ionised, the authors considered this option unlikely [48].

In 2005, Wang et al. [39] attempted to measure pH values of fatty acids in PG and determined that shorter chain fatty acids had lower pH levels in this solvent. The authors also found that the addition of PHY increased the pH values of given formulations, suggesting neutralisation of the acid, and thus ion pairing (values not disclosed by the authors).

**Figure 14:**
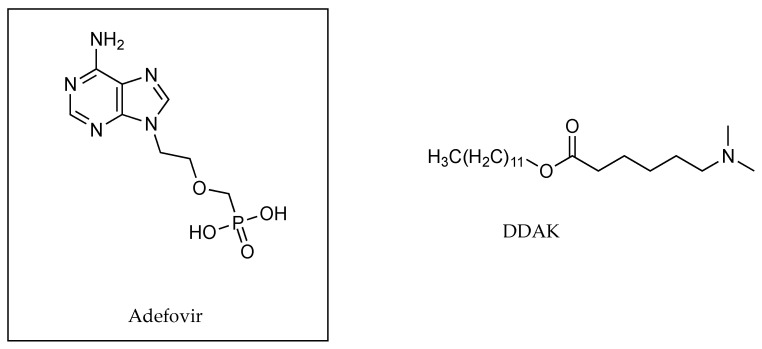
Adefovir and DDAK.

Vávrová and co-workers’ study in 2008 described the complex molecular structures of adefovir and its proposed counter ion, 6-(dimethylamino)hexanoate (DDAK), that had previously been used by this group as a permeation enhancer (Figure 14). The authors tested the permeation of the adefovir ion pair through porcine skin using phosphate buffer solutions. These solutions were adjusted using phosphoric acid and sodium hydroxide to various pH values ranging from 3.4 to 7.8 as shown below in Table 16. It was determined that the pH value of 5.8 was optimal for permeation experiments, with most of the adefovir taking the form of a hydrogenphosphonate monoanion with the tertiary amino group of DDAK being its main proton source [49].

#### 2.3.5. Counter Ion Concentration

The effects of adding excess amounts of counter ions to saturated solutions of their salt were considered by Pardo et al. [41] and are shown in Table 17. The consequences of increasing the amount of salicylate to achieve an 8:1 salicylate–PHY molar ratio in the donor compartment of a typical permeation study, caused a decrease in the solubility of the PHY-salicylate salt. Nonetheless, this had very little effect on the flux of PHY, that changed from 44.27 ± 9.16 to 48.40 ± 9.50 µmol cm^−2^ min^−1^. It did, on the other hand, increase the flux of salicylate approximately 4 fold, from 61.66 ± 2.54 to 247.30 ± 36.50 µmol cm^−2^ min^−1^.

Creating an excess instead, of PHY, resulted in the equivalent of a 6.5:1 molar ratio of PHY- salicylate in the donor solution. This change in proportion had no real impact on the flux of SA (61.66 ± 2.54 to 63.90 ± 7.00 µmol cm^−2^ min^−1^). It did, however, result in an increase in the flux of PHY by approximately 50% (44.27 ± 9.16 to 67.20 ± 7.70 µmol cm^−2^ min^−1^).

This relatively low increase in permeation when PHY concentration was increased, versus the lack of change in flux when PHY was decreased, might be explained by the ratio of PHY cations to salicylate anions. When the number of anions decreased, the PHY cations were more likely to bind to negatively charged groups present in the porcine membrane thus limiting their movement.

Vávrová et al. [49] also investigated the optimal quantity of the counter ion DDAK to be used in conjunction with adefovir, a zwitterionic drug used in the treatment of hepatitis B. This was done by maintaining the drug concentration at 2% and changing the amount of counter ion, DDAK, to 0, 0.5, 1, 2 and 3%. Permeation studies using porcine skin indicated optimal flux values at 1% for DDAK, at which point flux values plateaued. This was demonstrated by the different amounts of permeant that surpassed the skin barrier when maintaining the drug concentration at 2% and adjusting the amount of the counter ion, DDAK. With DDAK: 0%, flux: 0 µg cm^−2^ h^−1^; DDAK: 0.5%, flux: 14 µg cm^−2^ h^−1^, DDAK: 1%, flux: 27 µg cm^−2^ h^−1^, DDAK: 2%, flux: 26 µg cm^−2^ h^−1^ and when DDKA: 3%, flux: 26.5 µg cm^−2^ h^−1^.

**Figure 15:**
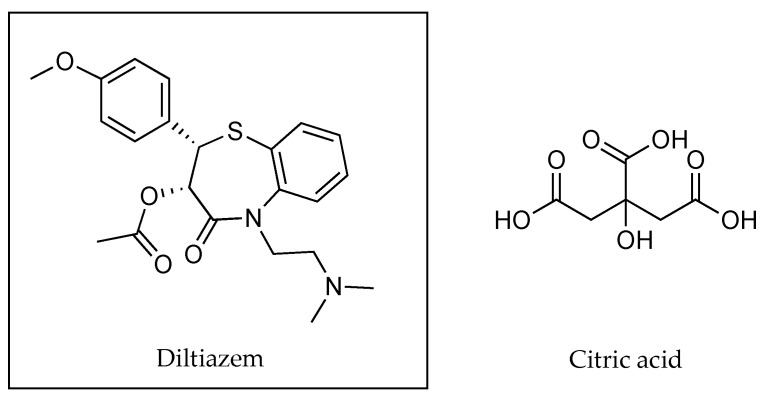
Diltiazem and citric acid counter ion.

In 2011, Chirio et al. [50] used citric acid as a counter ion to increase the lipophilicity of diltiazem at a 2% *w*/*w* concentration (as shown in Figure 15) in a thermosensitive gel formulation. The different formulations devised by the authors included a 1.6% *w*/*w* methylcellulose gel containing diltiazem either in the absence or presence of the counter ion in a 1:4 ratio (diltiazem: citric acid). Results from permeation studies using porcine skin showed that the flux of diltiazem increased from 1.8 ± 0.3 to 2.6 ± 0.5 µg cm^−2^ h^−1^ when applied with the counter ion. The skin accumulation of the active also increased from 85 ± 8 to 176 ± 11 µg cm^−2^ at 24 h. When the formulation was changed to a 16% *w*/*w* Pluronic F127 gel, flux values for diltiazem in the absence of the counter ion were shown to be 2.1 ± 0.4 µg cm^−2^ h^−1^, and 3.0 ± 0.5 µg cm^−2^ h^−1^ when the counter ion was included at a 1:1 ratio. Skin accumulation also increased from 77 ± 6 to 151 ± 12 µg cm^−2^ after a 24 h permeation study. The change of polymeric continuous phase of these gels resulted in a much lower concentration of counter ion being used to achieve similar permeation outcomes, demonstrating the importance of different molecular environments for ion pairing formation [50].

Due to the challenges involved in delivering sufficient quantities of drugs through the skin, the issue of toxicity is rarely addressed. It was, however, considered by Vávrová et al. [49] who specifically limited the amount of adefovir to a maximum concentration of 20 mg mL^−1^, in one set of experiments, despite drug solubility increasing up to as much as 120 mg mL^−1^ at different pH values. Fini et al. [32] also cautioned against the potential toxicity of the aliphatic amine counter ions used in their 2012 studies, as these do accompany the active when partitioning into the skin.

#### 2.3.6. Ion Pair and Penetration Enhancers

##### Penetration Enhancers Used as Ion Pairs

Green et al. [28] were the first to propose LA and OA as both counter ions and permeation enhancers in human skin studies in the late eighties. It was suggested that the enhanced flux of cationic naphazoline in conjunction with anionic fatty acids might be attributed to the increase in lipophilicity of naphazoline conveyed by ion pairing. This was confirmed by the results of PC studies shown previously in Table 1.

The counter ion, DDAK, used by Vávrová et al. [49] in conjunction with adefovir had previously been shown to be an effective permeation enhancer. This was demonstrated with pre treatment of porcine skin by the counter ion in advance of the application of the adefovir solution, with results highlighted in Table 18. When combined with adefovir in solution, the addition of 1% of DDAK significantly reduced the solubility of adefovir at its optimal pH, 5.8, from ~124 to ~71 mg mL^−1^. The reduction in solubility was of no consequence for this particular study, as the concentration was higher than that used in previous permeation experiments. This combination resulted in the flux increasing from ~16.5 to ~25.6 µg cm^−2^ h^−1^ in porcine skin experiments, when compared with pre-treatment alone. When experiments were conducted with human skin using adefovir alone, the authors observed that flux was “an order of magnitude lower” than it had been through porcine skin, but when applied in conjunction with DDAK it increased by 179 fold, from ~0.04 to 8.93 µg cm^−2^ h^−1^. As mentioned previously, the authors noted that at the chosen pH value of 5.8, much of the adefovir was in the form of a hydrogenphosphonate monoanion, whilst the tertiary amino group of DDAK was protonated. They suggested that it was likely therefore, that ion pairing contributed to the reduction in solubility, and to the increased flux of the drug.

##### Ion Pairs and the Inclusion of Penetration Enhancers

Auner et al. [36] combined the use of ALA and the counter ion, cetylpyridinium chloride (CP) at pH 7, with a permeation enhancer, 6-ketocholestanol (KC). The permeation enhancer was formulated into phosphatidylcholine liposomes. This combination increased the flux of ALA by 3.5 fold from ~6.26 to ~23.00 µg/cm^−2^ after a period of 4 h as shown in Table 19.

### 2.4. Ion Pairs and the Customisation of Drug Permeating Amounts

**Figure 16:**
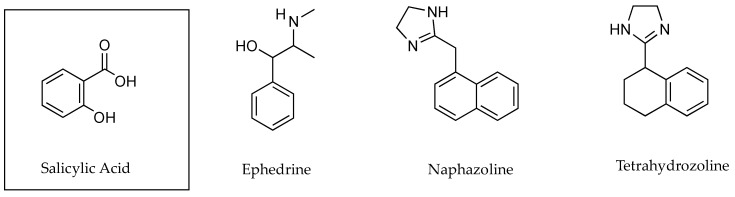
SA and counter ions ephedrine, naphazoline and tetrahydrozoline.

Ion pairs have been used in an attempt to increase drug flux through the skin. There are occasions, however, where the permeation of active ingredients into the systemic circulation is not desirable. Vasoconstrictors (VC) have been used as ion pairs in order to increase the residence time of drugs in the skin and local tissues by reducing dermal clearance [51]. Cross and co-workers tested three different VCs in a 1:1 molar ratio with SA (Figure 16) using liquid paraffin as a vehicle (Table 20). These formulations were then applied to a human abdominal epidermal membrane using conventional in vitro permeation methodology. Analysis of flux values as well as skin retention of the SA and VCs was then undertaken. It was found that following absorption into the membrane, there were unequal molar ratios of the VC and the SA in both the membrane and the receptor fluid. As shown in Table 20, there was greater epidermal flux and higher retention of the SA in conjunction with the VC, ephedrine. Flux of SA in combination with ephedrine was 18.6 ± 0.6 µg cm^−2^ h^−1^, with naphazoline was 7.8 ± 0.8 µg cm^−2^ h^−1^ and with tetrahydrozoline was 1.1 ± 0.1 µg cm^−2^ h^−1^. Skin retention of SA with ephedrine was 4.2 ± 0.7 µg mg^−1^; with naphazoline, it was 3.5 ± 1.1 µg mg^−1^; and with tetrahydrozoline, it was 2.8 ± 1.1 µg mg^−1^.

In a previously discussed study by Trotta that required the maximum concentration of the RA in the skin with minimal dermal clearance, valine methyl esters and phenylalanine ethyl esters were shown to increase the permeation of the active through porcine skin as shown in Table 21. When RA was used in conjunction with either of these counter ions in two different oil in water microemulsions, RA permeation was reduced considerably, from 0.13 ± 0.02 to less than 0.01 µg cm^−2^ h^−1^. There was also a corresponding increase in skin accumulation from a minimum amount of 1.0 ± 0.2 µg cm^−2^ when RA permeated alone, to 13.3 ± 2.10 µg cm^−2^ when combined with the phenylalanine ethyl esters in microemulsion (a) as shown in Table 21. The overall increase in localised delivery using the microemulsions with ion pairs was 4–5 fold that of RA alone. There was also a total reduction in flux [35].

**Figure 17:**
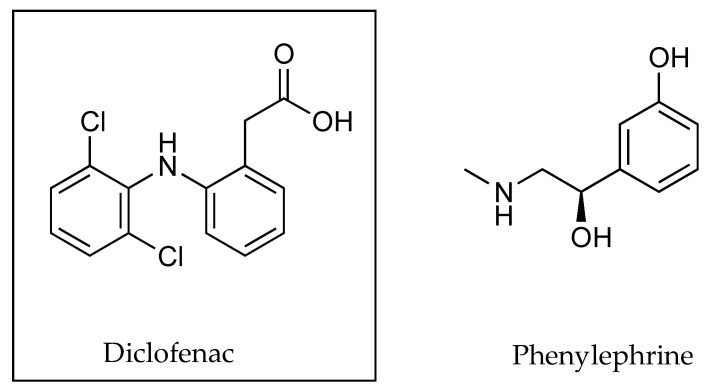
DIC and phenylephrine counter ion.

Another example of the use of a vasoconstrictor to enhance dermal accumulation was introduced by Uchino et al. [50] when combining DIC with phenylephrine as a counter ion (seen in Figure 17). After a 48 h in vitro permeation study, the cumulative amounts of DIC that had permeated after applying DIC, or the ion pair complex, to pig skin were 76.1 ± 26.6 and 118.9 ± 45.8 µg cm^−2^, respectively. The amounts of DIC found in the epidermis as a result of the same applications, were 3.34 ± 1.04 and 5.58 ± 1.43 µg mg^−1^, respectively. The concentrations of DIC in the dermis were recorded as 0.35 ± 0.09 and 0.45 ± 0.14 µg mg^−1^ for both neat DIC and the active when combined with phenylephrine as an ion pair [50,52].

An amino acid derivative, carnitine, has been investigated as a potential alternative exfoliator, due to the side effects of conventional exfoliation methods [40]. As calcium is important in the adhesion of corneocytes, a reduction in calcium ions in the skin reduces cohesion of the corneocytes, thus improving exfoliation. The calcium chelating abilities of carnitine were considered in a recent study by In et al. [40]. As an amphoteric molecule, penetration of carnitine has been regarded as extremely limited. A zwitterionic counter ion, hydrogenated soya phosphatidylcholine (HSC), was utilised to both increase partitioning into the skin and to limit permeation to the epidermis. The percentage of applied carnitine (4% *w*/*w*), found in the epidermal layers of porcine skin after a 24 h in vitro permeation study was approximately 9.13% when carnitine was applied without any counter ion, and 23.71% when applied with HSC. In this study, no permeation beyond the epidermis was seen, showing the potential of ion pairs in retention of skin compounds [40].

**Figure 18:**
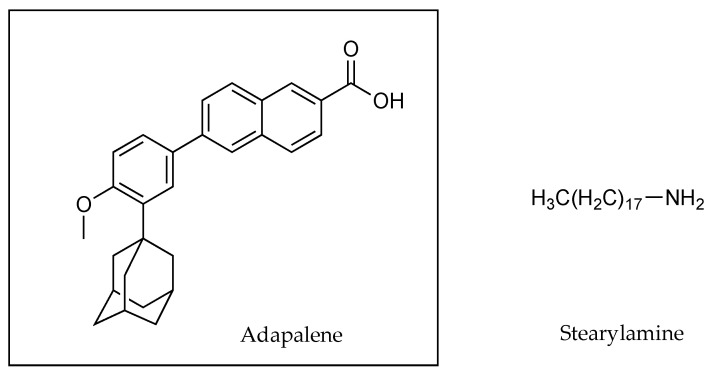
Adapalene and stearylamine counter ion.

Recently, ion pairing was used in a novel fashion by Rodrigues and co-workers to increase the lipophilicity of the hydrophobic drug, adapalene [53]. This molecule has been used for the topical treatment of acne. However, patient compliance is poor because of skin irritation. By encapsulating adapalene (0.1% *w*/*w*) in solid lipid nanoparticles (SLPs) in conjunction with stearylamine as a counter ion (Figure 18), several improvements in the formulation and delivery of adapalene were evident. According to the authors, crystals of adapalene were no longer observed in the external aqueous phase of the formulation as a result of efficient encapsulation due to ion pairing. While both compounds (i.e., adapalene and stearylamine) were considered practically insoluble in water, the interaction between these two molecular entities appeared to ensure that adapalene did not partition out of the lipid phase. Furthermore, the SLP formulation resulted in 4-fold more adapalene in the epidermis (16.36 ± 1.79 compared to 4.08 ± 0.22 µg cm^−2^) and 7-fold more retention in the dermis (1.17 ± 0.12 compared to 0.16 ± 0.03 µg cm^−2^), than the marketed gel formulation (0.1% *w*/*w* AD), in a porcine skin permeation study [53].

### 2.5. Kinetics

The organic ion pair, PHY–salicylate salt, was studied in human skin by Pardo et al. [41]. Since diffusion of the individual ion species occurred at different rates, it was concluded that while the diffusion of each component of the ion pair did follow Fick’s laws of diffusion, the rate at which they occurred was influenced by a number of factors. These included the pH of the membrane, the pK_a_ of the individual molecules and the charge bearing groups contained within the membrane. The authors suggested that the apparent net negative charge of the skin at physiological pH, could explain the slower permeation of the positively charged PHY.

In adjusting the molar ratios of the two species to determine the impact on permeation, the authors found that increasing salicylate to achieve an 8:1 salicylate—PHY molar ratio in the donor compartment of a typical permeation study, caused a decrease in the solubility of the PHY-salicylate salt. Nonetheless, this had very little effect on the flux of PHY, which changed from 44.27 ± 9.16 to 48.40 ± 9.50 µmol cm^−2^ min^−1^. It did, on the other hand, increase the flux of salicylate approximately 4 fold, from 61.66 ± 2.54 to 247.30 ± 36.50 µmol cm^−2^ min^−1^. Creating an excess instead, of PHY, resulted in the equivalent of a 6.5:1 molar ratio of PHY- salicylate in the donor solution. This change in proportion had no real impact on the flux of SA (61.66 ± 2.54. to 63.90 ± 7.00 µmol cm^−2^ min^−1^). It did, however, result in an increase in the flux of PHY by approximately 50% (44.27 ± 9.16 to 67.20 ± 7.70 µmol cm^−2^ min^−1^).

This relatively low increase in permeation when the PHY concentration was increased, versus no change in flux when PHY was decreased, was explained by the ratio of PHY cations to salicylate anions. When the number of anions decreased, the PHY cations were more likely to bind to negatively charged groups present in the porcine membrane thus limiting their movement. Conversely, the lack of positively charged groups in the membrane would facilitate the flux of the salicylate ion.

Other contributory factors in relation to the permeation kinetics of the permeation included solvent selection, combination and titration. The solvents IPA, with a ε of 18.62 at 20 °C [42,43], and IPM, with a ε of 3.31 at 25 °C [44], were mixed in various combinations. As shown in Table 9, it was found that a solvent mixture comprising a 70:30 ratio of IPA to IPM had the best impact on permeation. Flux of PHY increased from 0.56 ± 0.08 × 10^4^ µmol cm^−2^ m^−1^ at its lowest rate determined when the solvent comprised 100% of IPA, to 44.27 ± 9.16 × 10^4^ µmol cm^−2^ m^−1^ when the solvent mixture contained 70% IPA. Flux of salicylate increased from 1.47 ± 0.11 × 10^4^ µmol cm^−2^ m^−1^ at its lowest rate, which occurred when the solvent comprised 100% IPM to 61.66 ± 2.54 µmol cm^−2^ m^−1^ when the optimal ratio of IPA to IPM was 70:30 [41].

### 2.6. Ion Pairs in Marketed Formulations

A commonly used NSAID, DIC, is generally formulated as a salt to overcome the characteristic low solubility of the free acid. Salt forms such as sodium, epolamine (N-(2-hydroxyethyl)pyrrolidine) and diethylamine have been shown to partition from aqueous into lipid layers as ion pairs by Fini et al. [15,32]. These counter ions or salts are used in a number of commercial formulations as presented in Table 22. Although other formulations are available on the market, these are not included in the present document.

## 3. Conclusions

The literature relating to ion pairs has been examined and critically considered with the intention of elucidating common approaches used when employing ion pairing in the delivery of drugs via the skin. Not all factors influencing ion pairing, such as hydrogen bonding and solvation, were considered in the publications reviewed. Furthermore the areas identified, while examined individually, can not be considered in isolation.

PC studies have often been undertaken to determine the ability of ions to form ion pairs and these results have sometimes, but not always, translated into increased permeation results. While outcomes of regression analyses and QSARs have shown that the PC, as a measure of lipophilicity or hydrophobicity, is one of the most important predictors of flux, this is not always the case. This is unsurprising, given that permeation relies on both the partition and diffusion of molecules involved. Despite this, few studies considered the retention of the drug in the biological membranes in addition to flux, when considering the efficacy of PC experiments.

In terms of characteristics of the counter ion, experiments have confirmed that the ability to form ion pairs is impacted by the distance between centres of charge. Ions of a smaller radius, such as lithium, were better able to form ion pairs than those with a larger radius, such as caesium. Other observations relating to the type of counter ion, considered the degree of substitution of amine structures. The pairing efficiency of amine counter ions in conjunction with SA, in human skin permeation studies, was observed to be tertiary > secondary > primary > quaternary. Investigations also revealed organic cations to be more successful than inorganic structures in forming ion pairs. Furthermore, it was established that increasing the carbon chain length of the counter ion increased partitioning and permeation through the skin. This was attributed to increased lipophilicity of the ion pair when compared to their singular molecular constituents.

Authors have suggested that the contribution of ion pairing may be more than an electrostatic interaction. In such cases, the hydrocarbon moieties of the functional groups of counter ions may also contribute to masking or shielding the charges of the chemical composites. This conceptual experimental idea that longer alkyl chains could possibly increase such masking or shielding, would indeed explain the variety of permeation data shown by many authors.

General principles relating to solubility and the ability to partition or permeate have been analysed. The comparison of structurally related counter ions, showed very clearly that while those with hydroxy groups resulted in higher aqueous solubility, this did not ordinarily translate into a higher permeation coefficient. It was confirmed that solubility of a substance in its formulation does not ensure partition into a biological membrane. Furthermore, it was indicated that where solubility parameters of the solute and the solvent are too similar, it may be concluded that partitioning of the analyte into the membrane is not assured. Importantly, it is essential to bear in mind that ion pairing strategies are always subject to all factors affecting skin permeations.

As crucial components of semi-solid formulations, solvent systems with a reduced ε value are more likely to stabilise ion pairs. The impact of different solvent combinations on the permeation of counter ions was evaluated, as was the impact of different counter ions or different quantities of counter ions, on the conductivity of the solution. However, no information has been disclosed in recent studies regarding the conductivity of various solvent systems when utilising the same counter ion combinations.

The impact of pH has also been considered by many authors in ion pair research. Although the pH partition theory considers that only unionised drugs permeate the skin, it is clear that some ionised drugs are in fact able to partition when charges are masked.

The important issue of toxicity was addressed in only two of the papers reviewed, despite the significance of this matter when selecting potential counter ions. The authors may have given no consideration to this matter, due to their experiments being initial investigations into the ability of various ions to pair, and to the impact of different structures on this ability. However, this is an important concern that should be addressed in the selection process. Furthermore, the potential for skin related irritations did not appear to be a factor when choosing possible counter ions in any of the studies reviewed. As these formulations are to be applied topically, the likelihood of skin irritation must be contemplated. One such example is benzalkonium chloride that is considered an irritant when used at a concentration of 7.5% [54,55], thus use of this compound as a counter ion should be limited.

The concept of ion pairs has developed in novel ways. This approach has not only been explored to increase permeation through the skin, but to control or limit permeation to the membrane itself. Permeation enhancers have been used as ion pairs, and ion pairs have been used in conjunction with permeation enhancers.

Ion pairs have also been used to improve the stability of solid lipid nanoparticle formulations. Thus, the formation of ion pairs is a versatile strategy, and one to be considered when formulating actives for topical and transdermal delivery. They should not be examined in isolation, because as noted, they are subject to all the factors that impact the application of drugs to or via the skin. In future investigations, it would be interesting to consider not only the impact of the solvent ε value on the conductivity of the formulation, but also to evaluate the importance of solubility parameters of analytes and solvents used for such studies. This concept could potentially result in a formulation from which counter ions would be more likely to partition into the membrane. Additionally, mass balance studies combined with conventional permeation studies should provide additional information relating to the distribution of the pharmaceutical ingredient, bearing in mind the impact of the solubility parameters of the membrane in relation to the movement of the permeant.

## Figures and Tables

**Table 1:** Impact of OA and LA on the PC and permeability coefficient values for naphazoline. Concentration of naphazoline applied was 0.05 M (*n* = 4, values represent the mean ± S.D.) Adapted with permission from [28], Elsevier, 1988.

**Partition** **Coefficient of naphazoline**	Isopropyl myristate (IPM)/buffer (pH 7.4)	0.02 ± 0.01
	OA in IPM/buffer (pH 7.4)	0.36 ± 0.04
	LA in IPM/buffer (pH 7.4)	0.45 ± 0.06
	IPM/buffer (pH 8.0)	0.03 ± 0.01
**Permeability coefficient of naphazoline using human skin** **(cm h^−1^ × 10^−3^)**	Naphazoline HCl	~0.33
	Naphazoline plus OA	~2.17
	Naphazoline plus LA	~2.58

**Table 2:** PC and flux for lignocaine HCl at different donor pH values. For flux experiments, 2% lignocaine hydrochloride was applied in buffer solutions (*n* = 3, values represent the mean ± S.D.) Adapted with permission from [29], Elsevier, 2000.

pH	PC (Calculated)	Flux (µg cm^−2^ h^−1^)
4.0	~0.19	1.2 ± 1.2
5.5	~0.40	13.0 ± 2.0
7.0	~6.76	118.0 ± 30.0

**Table 3:** PC, unionised fractions, permeability coefficient and flux (across human skin) for benzydamine HCl at different donor pH values. For flux experiments, 2% benzydamine hydrochloride was applied in buffer solutions (*n* = 6, values represent the mean ± S.D.) Adapted with permission from [30], Elsevier, 2005.

pH	PC ~	Fraction Unionised	Permeability Coefficient (cm h^−1^)	Flux (µg cm^−2^ h^−1^)
5.0	1.62	6.3 × 10^−3^	2.6 × 10^−4^	5.13 ± 2.42
6.0	5.75	0.0631	3.8 × 10^−3^	39.07 ± 10.5
7.0	28.18	0.627	6.1 × 10^−2^	269.09 ± 58.5

**Table 4:** Effect of tertiary amines on the PC and the flux of SA through human epidermis. Donor phase comprised equimolar concentrations of salicylate anion and amine counter ion, with actual concentration of SA not provided by the authors; donor solvent: ethanol to propylene glycol (2:1 *v*/*v*) (*n* ≥ 3, values represent the mean ± S.E.M.) Adapted from [31], Wiley, 2000.

SA with Counter Ion:	PC in Octanol-Phosphate Buffer (pH 5.0)	Flux (mg cm ^−2^ h ^−1^ × 10 ^−2^)
Triethanolamine	0.007 ± 0.00	11.90 ± 1.23
Triethylamine	0.360 ± 0.00	15.40 ± 3.85
Tripropylamine	3.180 ± 0.04	18.50 ± 2.26
Tripentylamine	109.77 ± 11.37	19.50 ± 3.63
Trihexylamine	152.17 ± 26.81	22.60 ± 1.14
Trioctylamine	140.58 ± 16.33	27.90 ± 3.98
Tridodecylamine	140.66 ± 17.23	42.70 ± 2.04

**Table 5:** Permeation parameters of DIC salts. According to the authors, saturated solutions were applied in permeation studies (for solubility and partition experiments, *n* = 3; for permeation experiments used to calculate permeation coefficient *n* ≥ 5; values represent the mean) Adapted from [32], MDPI, 2012.

DIC Plus Counter Ion:	PC	Solubility (µg cm^−3^ × 10^3^)	Permeation Coefficient (cm h^−1^ × 10^3^)
Monoethanolamine	1.20	9.9	0.70
Monoethylamine	1.02	6.1	2.00
Diethanolamine	1.20	18.0	2.80
Diethylamine	1.48	13.7	3.70
Triethanolamine	4.37	3.4	3.00
Triethylamine	7.08	6.7	3.40
N-2-hydroxyethyl pyrrolidine	1.48	20.2	9.60
Pyrrolidine	1.62	2.0	21.00
N-2-hydroxyethyl piperidine	1.95	10.7	7.70
Piperidine	9.33	4.3	20.00
N-2-hydroxyethyl morpholine	10.96	4.4	4.80
Morpholine	2.24	6.9	3.80
N-2-hydroxyethyl piperazine	1.74	12.5	13.00
Piperazine	4.68	0.4	45.00

**Table 6:** PC and cumulative amount permeated of pure ALA and in conjunction with various counter ions at pH 4.0 and pH 7.0. The concentration of ALA applied in the buffer solutions was 0.4 mg mL^−1^ (for permeation experiments *n* ≥ 3, values represent the mean ± S.D.) Adapted with permission from [36], Elsevier, 2003.

ALA Plus Counter Ion	pH	PC ~(×10^−1^)	Cumulative Amount of ALA ~(µg cm^−2^) after 4 h Using Porcine Skin
None	4.0	1.20	5.11
Sodium-1-octanesulfonic acid	4.0	2.14	10.7
Sodium-1-heptanesulfonic acid	4.0	4.68	10.0
Sodium-1-pentanesulfonic acid monohydrate	4.0	2.69	10.0
None	7.0	1.51	6.5
Cetylpyridinium chloride	7.0	9.12	11.0
Cetyltrimethylammonium bromide	7.0	8.13	5.0
Benzalkonium chloride	7.0	6.03	7.0

**Table 7:** Impact of tertiary amines on conductivity, on the permeation of SA through human epidermis, plus physicochemical properties of tertiary counter ions (for permeation experiments *n* ≥ 3 ≤ 6, values represent the mean ± S.E.M.) Adapted from [31], Wiley, 2000.

Salicylic Acid Plus Counter Ion	Molecular Weight of Counter Ion (Da)	Molal Volume of Counter Ion (cm^3^ mol^−1^)	Flux (mg cm^−2^ h^−1^) × 10^−2^	Conductivity (mS cm^−1^)
None	-	-	8.90 ± 1.20	2.03 ± 0.06
Triethylamine	101.20	108.00	15.40 ± 3.85	1.77 ± 0.06
Tripropylamine	143.27	156.60	18.50 ± 2.26	1.35 ± 0.06
Tripentylamine	227.44	253.80	19.50 ± 3.63	0.90 ± 0.00
Trihexylamine	269.52	302.40	22.60 ± 1.14	0.80 ± 0.00
Trioctylamine	353.68	399.60	27.90 ± 3.98	0.60 ± 0.00
Tridodecylamine	522.00	592.50	42.70 ± 2.04	0.30 ± 0.00

**Table 8:** PC values, solubility and flux values for propranolol (PR) racemate (RS), propranolol S-enantiomer (S-PR), individually and in conjunction with benzoate (Bz) and oleate (Ol) counter ions. According to the authors, saturated solutions were applied. Solubility values for (RS)_-PR-Ol and (S)-PR-Ol were assumed to be 0.02 mg mL^−1^, the concentration determined to be their critical micellular concentration (CMC). For solubility and permeation experiments *n* = 3, flux values represent the mean ± S.D. Adapted with permission from [33], Elsevier, 2010.

Counter Ions	PC Value ~	Solubility Saline (mg mL^−1^)	Flux Saline (µg cm^−2^h^−1^)	Solubility MO (mg mL^−1^)	Flux MO (µg cm^−2^ h^−1^)
(RS)-PR	10.00	0.189	18.0 ± 5.1	1.321	24.2 ± 4.7
(S)-PR	9.33	0.432	44.7 ± 5.1	3.111	41.9 ± 1.5
(RS)-PR-Bz	11.74	4.430	1.7 ± 0.2	0.065	1.6 ± 0.3
(S)-PR Bz	11.48	9.560	3.0 ± 0.4	0.118	2.9 ± 0.7
(RS)-PR-Ol	17.78	0.020	7.0 ± 1.4	3.930	7.3 ± 1.4
(S)-PR-Ol	19.05	0.020	6.2 ± 0.9	5.874	11.1 ± 1.7

**Table 9:** Flux values for PHY and salicylate through excised human skin, which according to the authors, were delivered from equimolar saturated solutions of solvents consisting of IPA, IPM and their mixtures (*n* ≥ 3 ≤ 8, values represent the mean ± S.E.M.) Adapted from [41], Wiley, 1992.

Volume Fraction of IPA in IPA-IPM Solvent Mixture	Flux × 10^4^ µmol cm^−2^ m^−1^	
	PHY	Salicylate
0	1.28 ± 0.35	1.47 ± 0.11
0.1	5.75 ± 0.58	6.3 ± 0.50
0.3	14.55 ± 0.71	18.2 ± 0.56
0.5	31.70 ± 3.10	47.8 ± 11.50
0.7	44.27 ± 9.16	61.66 ± 2.54
0.9	5.52 ± 0.28	17.70 ± 1.90
1	0.56 ± 0.08	1.87 ± 0.10

**Table 10:** Impact of different solvents on the solubility and flux of DIC in conjunction with different salts as counter ions. According to the authors, saturated solutions of the DIC salts were applied for permeation experiments (*n* = 3, values represent the mean ± S.D.) Adapted with permission from [38], Elsevier, 2007.

DIC Plus Counter Ion	Parameter	Solvents			
		Water	PG	TC^®^	OA
Sodium	Solubility (µg mL^−1^)	37 ± 10	567 ± 31	660 ± 70	25 ± 10
	Flux (µg cm^−2^ h^−1^)	2.29 ± 0.37	1.21 ± 0.06	0.06 ± 0.01	1.84 ± 0.18
Potassium	Solubility (µg mL^−1^)	218 ± 80	898 ± 79	709 ± 52	60 ± 50
	Flux (µg cm^−2^ h^−1^)	1.35 ± 0.72	0.04 ± 0.02	0.84 ± 0.06	1.17 ± 0.17
Diethylamine	Solubility (µg mL^−1^)	19 ± 10	384 ± 14	279 ± 10	63 ± 60
	Flux (µg cm^−2^ h^−1^)	5.60 ± 2.14	0.35 ± 0.04	0.96 ± 0.59	2.74 ± 0.94
Epolamine	Solubility (µg mL^−1^)	557 ±15	637 ± 60	430 ± 0.00	94 ± 70
	Flux (µg cm^−2^ h^−1^)	2.90 ± 0.91	0.46 ± 0.21	0.03 ± 0.00	3.11 ± 0.18

**Table 11:** Flux, conductivity (Con) and solubility (Sol) of PHY in PG and MO. According to the authors, saturated drug solutions were used in permeation experiments. Fatty acid counter ions are indicated according to length of carbon chain. 18:1 (OA, 1 double bond) and 18:2 (LA, 2 double bonds) (for conductivity and permeation experiments *n* = 3, values represent the mean ± S.D.) Adapted with permission from [39], Elsevier, 2005.

PHY Plus Fatty Acid Counter Ion:	Flux (PG) (µg cm^−2^ h^−1^)	Con ~ (µS cm^−1^) PG	Sol (mg mL^−1^)	Flux (MO) (µg cm^−2^ h^−1^)	Con ~ (µS cm^−1^) MO ×10^−2^	Sol (mg mL^−1^)
None	-	0.25	71.0	2.5 ± 0.7	9.25	1.7
2	-	16	-	-	9.45	-
3	-	14	-	-	8.65	-
8	-	10	116.0	3.2 ± 0.6	9.05	5.1
10	-	9.5	80.7	6.6 ± 2.3	9.40	10.8
12	0.2 ± 0.0	8	88.7	14.0 ± 3.0	8.95	15.3
18:1	19.7 ± 8.2	3	83.9	13.9 ± 7.1	8.80	15.9
18:2	2.4 ± 0.9	4	85.3	3.9 ± 1.1	9.15	15.7

**Table 12:** Conductance of potassium iodide in methanol at different temperatures. Units were not provided, but the trend in overall values may be used to demonstrate the concepts. Adapted from [19], American Chemical Society, 1956. The bold indicates the temperature of maximum conductance.

~Temperature °C	~Conductance
80	500 × 10^6^
100	576 × 10^6^
120	638 × 10^6^
**150**	**700 × 10^6^**
180	654 × 10^6^
200	577 × 10^6^
220	400 × 10^6^
240	14.2 × 10^−2^

**Table 13:** The temperature at which the highest conductance value is reached for potassium iodide in solvents of different ε values: methylamine, ammonia and methanol. Adapted from [19] American Chemical Society, 1956.

Solvent	ε	Conductance Maximum Reached at °C
Methylamine	9.4 at 25 °C	15
Ammonia	22.4 at ~33.3 °C	25
Methanol	32.8 at 25 °C	150

**Table 14:** Flux ± standard deviation of 14.48 mM solutions of SA across human skin at a range of pH values (experiments *n* = 2, values represent the mean ± S.E.) Adapted with permission from [47], Elsevier, 2000.

pH	Flux (µmol cm^−2^ h^−1^)
2.10	0.72 ± 0.057
2.27	0.59 ± 0.016
2.72	0.54 ± 0.009
3.13	0.25 ± 0.006
3.50	0.15 ± 0.005
3.90	0.07 ± 0.001
4.30	0.05 ± 0.001
4.71	0.04 ± 0.000
5.13	0.01 ± 0.000

**Table 15:** Apparent permeability coefficients for different pH values for lidocaine HCl and in the presence of L-TC and O-TC. According to the authors, saturated solutions of lidocaine were used (*n* = 6, values represent the mean ± S.D.) Adapted with permission from [48], Elsevier, 2005.

pH	Lidocaine HCl	Lidocaine Plus 2% L-TC	Lidocaine Plus 2% O-TC
	Permeability Coefficient ×10^4^ cm h^−1^	Permeability Coefficient ×10^4^ cm h^−1^	Permeability Coefficient ×10^4^ cm h^−1^
5.0	1.55 ± 0.31	18.46 ± 5.60	5.83 ± 0.65
7.0	1.81 ± 1.1	19.58 ± 3.82	6.24 ± 0.31
9.0	5.65 ± 1.52	3.34 ± 0.22	15.08 ± 2.30

**Table 16:** Flux and skin concentration of 2% adefovir with and without DDAK though porcine skin plotted against pH (*n* = 4 for all experiments except pH 5.8 where *n* = 12, values represent the mean). Adapted from [49], Elsevier, 2008.

pH Values		3.4	3.8	4.8	5.8	6.8	7.8
Flux of Adefovir ~ (µg cm^−2^ h^−1^)	2% Adefovir	0.2	1	2	1	3	4
	2% Adefovir + 1% DDAK	9.5	10	18	27	26.5	10
Adefovir Skin Concentration ~(µg g^−1^)	2% Adefovir	103	221	235	191	191	221
	2% Adefovir + 1% DDAK	412	538	708	771	693	412

**Table 17:** Effect of solute composition on concentration (C_v_), flux values and permeability coefficients of PHY and salicylate, when delivered through human skin (*n* ≥ 3 ≤ 8, values represent the mean ± S.E.M.). Adapted from [41], Wiley, 1992.

PHY: SA Ratio	Flux × 10^4^ (µmol cm^−2^ min^−1^)		Permeability Coefficient × 10^4^ (cm min^−1^)		C_v_ µmol cm^−3^	
	PHY	salicylate	PHY	salicylate	PHY	salicylate
1:1	44.27 ± 9.16	61.66 ± 2.54	1.86 ± 0.39	2.60 ± 0.10	23.70 ± 0.55	23.70 ± 0.55
1:8	48.40 ± 9.50	247.30 ± 36.50	3.25 ± 0.64	2.10 ± 0.30	14.90 ± 0.40	120.00 ± 15.20
6.5:1	67.20 ± 7.70	63.90 ± 7.00	0.44 ± 0.05	2.68 ± 0.30	153.40 ± 7.70	23.70 ± 3.40

**Table 18:** Flux and skin concentration of adefovir after use of DDAK (for human skin experiments *n* ≥ 3 ≤ 4, except for 2% adefovir samples where *n* = 12, for porcine skin experiments *n* = 4, except for co-application samples where *n* = 12, values represent the mean). Adapted from [49], Elsevier, 2008.

Membrane	Application	Flux ~(µg cm^−2^ h^−1^)	Skin Concentration (Epidermins Incl SC for Human Skin)~
Porcine skin	Co-application of 2% adefovir, 1% DDAK	25.6	31.3 µg g^−1^
	2% adefovir pre-treatment of skin with DDAK	16.5	17.1 µg g^−1^
Human skin	Co-application of 2% adefovir, 1% DDAK	8.93	21.30 µg cm^−2^
	2% adefovir no DDAK	0.04	5.72 µg cm^−2^

**Table 19:** Permeation of ALA through porcine skin with unloaded phosphatidylcholine liposomes; with CP counter ion and unloaded phosphatidylcholine liposomes and with CP counter ion and KC loaded phosphatidylcholine liposomes. The concentration of ALA applied in the buffer solutions was 0.4 mg mL (*n* ≥ 3 values represent the mean). Adapted from [36], Elsevier, 2003.

ALA with:	pH	Cumulative amount of ALA ~(µg/cm^−2^) after 4 h Using Porcine Skin
counter ion CP plus phosphatidylcholine liposomes loaded with KC	7.0	23.00
counter ion CP plus unloaded phosphatidylcholine liposomes	7.0	11.25
unloaded phosphatidylcholine liposomes	7.0	6.25

**Table 20:** Epidermal flux and tissue retention of VCs and SA following application to human skin. Concentrations applied were 10% *w*/*v* of equimolar amounts of SA and VC (*n* = 6, values represent the mean ± S.E). Used with permission from [51], Springer Nature, 2003.

VC	Flux (µg cm^−2^ h^−1^)		Skin Retention (µg mg^−1^)	
	VC	SA	VC	SA
Ephedrine	11.5 ± 2.3	18.6 ± 0.6	10.0 ± 0.4	4.2 ± 0.7
Naphazoline	12.0 ± 1.6	7.8 ± 0.8	20.7 ± 6.0	3.5 ± 1.1
Tetrahydrozoline	2.9 ± 0.5	1.1 ± 0.1	3.7 ± 0.6	2.8 ± 1.1

**Table 21:** Skin accumulation and flux of RA alone and in combination with various counter ions. Formulation used for non-microemulsion studies was a 0.005% (*w/w*) RA in ethanol–pH 6.4 buffer solution (1:2 *v/v*), in the absence or presence of counter ions in a 1: 50 molar ratio. Microemulsions contained 0.05% RA in the absence or presence of counter ions in a 1: 50 molar ratio. Microemulsion (a) comprised 56.7% water, 8.8% IPM, 5.3% Epikuron, 13.2% Oramix and 16.0% ethanol; microemulsion (b)) comprised 64.3% water, 10.0% IPM, 11.0% Epikuron, 13.6% Oramix and 9.2% ethanol (*n* ≥ 3, values represent the mean ± S.D.). Adapted with permission from [35], Elsevier, 2003.

RA with:	Skin Accumulation after 24 h (µg cm^−2^)	Flux (µg cm^−2^ h^−1^)
alone	1.0 ± 0.2	0.13 ± 0.02
tryptophan methyl ester hydrochloride	2.3 ± 0.6	0.19 ± 0.02
phenylalanine ethyl ester hydrochloride	3.4 ± 0.6	0.23 ± 0.03
valine methyl ester hydrochloride	3.7 ± 0.8	0.21 ± 0.03
Formulated as microemulsions oil in water microemulsions (a) and (b):		
(a) alone as microemulsion	3.3 ± 0.50	0.05 ± 0.01
(a) phenylalanine ethyl ester hydrochloride	13.3 ± 2.10	<0.01
(a) valine methyl ester hydrochloride	8.7 ± 1.6	<0.01
(b) alone as microemulsion	2.3 ± 0.50	0.04 ± 0.01
(b) phenylalanine ethyl ester hydrochloride	12.6 ± 1.8	<0.01
(b) valine methyl ester hydrochloride	10.8 ± 1.5	<0.01

**Table 22:** Commercial applications of DIC and counter ions sodium, epolamine and diethylammonium.

Commercial Name	Active	Ion Pair	Owner
Diclofenac 1% gel	DFA	Sodium	A A H Pharmaceuticals Ltd.
Diclofenac 1% gel	DFA	Sodium	Actavis UK Ltd.
Diclofenac 1% gel	DFA	Sodium	Alliance Healthcare (Distribution) Ltd.
Diclofenac 1% gel	DFA	Sodium	Typharm Ltd.
Diclofenac sodium topical gel 1%	DFA	Sodium	AvKARE
Diclofenac sodium topical gel 3%	DFA	Sodium	Taro
Diclofenac sodium topical solution (1.5%)	DFA	Sodium	Sola Pharmaceuticals
Flector gel 1%	DFA	Epolamine, sodium	Laboratoires Genevrier
Flector EP 10 mg/g	DFA	Epolamine	Medichemie
Flector tissugel 140 mg (medicated plaster)	DFA	Sodium	Windzor Pharma
Flector (patch)	DFA	Epolamine	Pfizer
Pennsaid ^®®^ (2% solution)	DFA	Sodium	Horizon Medicines LLC
Solaraze 3% gel	DFA	Sodium	Almirall Ltd.
Solacutan 3% gel	DFA	Sodium	Mibe Pharma UK Ltd.
Voltarol^®®^ joint pain relief 2.32% gel	DFA	diethylammonium	GSK
Voltarol^®®^ joint 12 h joint pain relief 2.32% gel	DFA	diethylammonium	GSK
Voltarol^®®^ osteoarthritis joint pain relief 1.16% gel	DFA	diethylammonium	GSK
Voltarol^®®^ back and muscle pain relief 1.16% gel	DFA	diethylammonium	GSK
Voltarol^®®^ 140 mg medicated plaster	DFA	Sodium	GSK

## Data Availability

Not applicable.
